# Lactate: A regulator of immune microenvironment and a clinical prognosis indicator in colorectal cancer

**DOI:** 10.3389/fimmu.2022.876195

**Published:** 2022-08-26

**Authors:** Daoqi Zhu, Yiping Jiang, Huihui Cao, Jiabin Yang, Yuqi Shu, Haowei Feng, Xiaoyu Yang, Xiaomin Sun, Meng Shao

**Affiliations:** ^1^ School of Traditional Chinese Medicine, Southern Medical University, Guangzhou, China; ^2^ Department of Pharmacy, Zhuhai People’s Hospital (Zhuhai hospital affiliated with Jinan University), Zhuhai, China; ^3^ Traditional Chinese Pharmacological, Third Level Research Laboratory of State Administration of Traditional Chinese Medicine, School of Traditional Chinese Medicine, Southern Medical University, Guangzhou, China; ^4^ Guangdong Provincial Key Laboratory of Chinese Medicine Pharmaceutics, School of Traditional Chinese Medicine, Southern Medical University, Guangzhou, China

**Keywords:** lactate score, colorectal cancer, immunotherapy, prognostic model, microenvironment

## Abstract

Lactate can play an immunosuppressive role in the tumor microenvironment and promote tumor development by recruiting and inducing the activity of immunosuppressive cells and molecules. High lactate concentrations are important for tumor cell metastasis, angiogenesis, and treatment resistance. With the in-depth studies on tumor metabolism, lactate, one of the key factors involved in glycolysis, has been increasing emerged its characteristic clinical value in colorectal cancer (CRC). In this study, lactate genes were screened based on lactate metabolism pathways. Subsequently, the lactate subtypes were determined by clustering and analysis of the subtypes at all levels, including immune checkpoints, immune infiltration, and clinical characteristics, which revealed the biological significance of lactate metabolism in CRC. Subtype-based differential gene analysis resulted in a lactate score, which stratifies the prognosis of CRC. We discovered that 27 lactate genes and 61 lactate-phenotype genes are associated with immune cell infiltration and have a significant prognostic efficacy. The CRC patients were clustered into four subtypes and five clusters, based on lactate genes and lactate-phenotype genes, respectively. There are significant differences in survival time and activities of hallmark pathways, namely immune-related signatures and chemokines, among these subtypes and clusters. Particularly, cluster 2 and subtype 1 have significantly higher lactate scores than that of the others. In conclusion, lactate score is an independent prognostic factor for cancer that can be used as a clinical guide for predicting CRC progression and as an evaluation factor for the effect of immunotherapy in CRC.

## Introduction

Colorectal cancer (CRC) is the third leading cause of cancer-related deaths worldwide, and it ranks second in the United States (1). In fact, the incidence of CRC is high in the western world, largely due to the modifiable environmental risk factors characterizing westernization, including obesity, physical inactivity, and alcohol and tobacco consumption (2). Since the pathogenic site of CRC is closely related with the intestinal microbial composition, a dysbiosis may increase the risk for developing CRC (3). Incidentally, the close association of the intestinal microbiotas with immune infiltration affects the tumor microenvironment (TME); hence, these microbes play important roles in tumor immunotherapy, especially programmed death-ligand 1 (PD‐L1) ‐related therapies (4). Regarding genetic factors, DNA mismatch repair genes, such as MutL homolog 1 (*MLH1*), MutS homolog 2 (*MSH2*), MutS homolog 6 (*MSH6*), and PMS1 homolog 2 (*PMS2*), and inherited germ-line mutations of the adenomatous polyposis coli (*APC*) gene play a key role in predisposing individuals to CRC (5, 6). However, environmental factors have a stronger influence than genetic factors in the pathogenesis of CRC, such as smoking, alcohol, obesity, and long-term intake of red or processed meat may increase risk for CRC (2). Drug resistance and desensitization to checkpoint inhibitors suggest that there is a dynamic interaction with the TME, which dictates the therapeutic outcome of CRC (7). This is precisely due to the complexity of the factors related to the CRC TME. Therefore, it is important to determine an accurate and valid indicator that can predict the therapeutic response and prognosis of CRC patients.

Glucose uptake and lactate accumulation are also gradually increased in tumor cells in the presence of normal oxygen content. Utilize glycolysis as the main source of energy metabolism to obtain a higher sugar-breaking capacity, allowing the conversion of glucose to lactate to generate adenosine triphosphate (ATP) (8). Recently, an increasing number of studies have indicated that the TME, including lactate as well as immune microenvironments, affects the progression of CRC as well as its clinical outcomes (9, 10). Incidentally, tumor cells exhibit an aberrant increase in glycolysis, which generates a high concentration of lactic acid that is necessary for malignant proliferation. Hence, a highly active aerobic glycolytic metabolism is one of the vital characteristic features of CRC cells. In fact, various key molecules of the glycolytic pathway, especially lactate dehydrogenase (LDH), are highly expressed in CRC, thereby driving the continuous acceleration of the glycolytic metabolism. Moreover, the increased expression of LDHs is often associated with the poor prognosis, poor progression-free survival (PFS), poor overall survival (OS), and local recurrence of the disease after radiotherapy (11). For instance, the abnormal expression of lactate dehydrogenase A (LDHA) is closely related to the differentiation and distant metastasis of CRC (12). Reportedly, after 12 weeks of first-line therapy, patients with high plasma LDHA levels have a poor prognosis and PFS (13). Furthermore, long noncoding RNAs (lncRNAs) affect the glycolytic pathway of CRC cells *in vitro* and *in vivo*. Wang et al. revealed that the insulin-like growth factor 2 mRNA-binding protein 2 (IGF2BP2) is stabilized by the long intergenic noncoding RNA for IGF2BP2 stability (LINRIS) *via* the ubiquitination-autophagy pathway, thereby inhibiting the CRC cell proliferation (14). Additionally, the glycolysis-associated lncRNA of colorectal cancer (GLCC1) promotes metabolic reprogramming of CRC cells through the heat-shock protein 90/cellular myelocytomatosis oncogene/LDHA (HSP90/c-Myc/LDHA) axis (15).

The increasing number of studies on lactate metabolism in CRC indicate that lactate may be a potential biomarker of CRC prognosis. In addition to directly promoting the malignant growth of tumors, lactate in the TME can also promote the evasion of immune responses by tumors *via* inhibition of proliferation and activity of immune cells. Lactate also promotes bone metastasis from CRC through the phosphatidylinositol 3-kinase (PI3K)-protein kinase B (AKT) pathway in CD115(+) precursors (16). Furthermore, lactate inhibits the proliferation of cytotoxic T lymphocytes (CTLs) as well as their cytokine production by > 95% and leads to a 50% decrease in cytotoxic activity (17). Tumor-derived lactate has a significant role in the mechanism of communication between macrophages and their “client cells,” particularly by stimulating the M2-like polarization of the tumor-associated macrophages (TAMs) (18). In fact, the tumor-derived lactate also inhibits the differentiation of monocytes into dendritic cells (DCs) (19) and leads to the generation of tolerogenic DCs through multiple mechanisms (20, 21). Meanwhile, lactate significantly reduces the cytotoxic activity of natural killer (NK) cells by downregulating the activating receptor NKp46 (22). Incidentally, PD-L1 functions predominantly in lactate microenvironments. Because of G protein-coupled receptor 81 (GRP81) of tumor cells will be activated in the lactate environment, then inhibit PKA, induce PD-L1 expression and therefore resulting in immunosuppression (23). Hence, lactate has a vital role in regulating the immune microenvironment in solid tumors. Even though the incidence of the lactate metabolism in CRC has been determined, the immune cell modulation effect of lactate on CRC needs to be examined. Therefore, the aim of this study is to construct a lactate score module and evaluate the efficacy of immunotherapy in CRC.

## Materials and methods

### Study population and datasets

The Cancer Genome Atlas (TCGA) colon adenocarcinoma (COAD) cohort (n = 594), including the genomic mutation, copy number variation, and transcriptome datasets, as well as the related clinical phenotypes, was downloaded from cBioPortal (cBioPortal, RRID : SCR_014555) (**Table S1**). This cohort contained the genomic mutation and copy number variation (CNV) datasets, the transcriptome dataset and clinical phenotypes including age, sex, American Joint Committee on Cancer (AJCC) tumor stage, and the tumor, nodes, and metastases (TNM) stage (https://www.cbioportal.org/study/summary?id=coadread_tcga_pan_can_atlas_2018). We also obtained information regarding the OS and PFS for further analysis. Another CRC transcriptome dataset (GSE41258, n = 240) along with the relevant clinical information was obtained from the Gene Expression Omnibus (GEO, RRID : SCR_005012). Moreover, we used the R package IMvigor210CoreBiologies to extract a urothelial carcinoma cohort (n = 298) treated with the PDL-1 inhibitor atezolizumab.

### Screening for lactate-related genes

After filtering the gene count matrix and eliminating the genes that were expressed in less than five samples, we used the R package DESeq2 to perform differential expression analysis. By searching with the keywords “lactic acid” and “lactate” in the MSigDB (V7.4), 13 lactate-related pathways were obtained (**Table S2**). Totally 245 genes have integrated from 13 lactate-related pathways by removing duplications and were defined as lactate genes. Among them, the genes with a fold change > 2 and false discovery rate (FDR) < 0.01 (Benjamini-Hochberg correction) between tumor samples and adjacent tissues were defined as lactate-related genes.

### Consistent clustering analysis and identification of differentially expressed genes

We used the R package ConsensusClusterPlus (ConsensusClusterPlus, RRID : SCR_016954) for consistent clustering analysis and DESeq2 (DESeq, RRID : SCR_000154)for identification of differentially expressed genes (DEGs). To determine the optimal number of clusters, we evaluated the results using cumulative distribution function and incremental area analysis with different clusters (2–10). The DEGs with fold change > 1.5 and FDR < 0.05 were defined as subtype-specific. The COAD patients were first clustered into four lactate-related subtypes (subtypes 1 - 4), based on lactate-related genes. Thereafter, 61 DEGs in subtypes 2 and 3 were identified as lactate phenotype-related genes by intersection. Finally, the COAD patients were re-clustered into five lactate phenotype-related subtypes (clusters 1 - 5), based on the lactate phenotype-related genes. We visualized the clustering results using the R package pheatmap, along with age, sex, AJCC tumor stage, and TNM stage.

### Development of a lactate-based signature for prognosis and validation

We subjected the 61 lactate phenotype-related genes to further screening for prognostic markers. Using univariate Cox regression analysis, we identified 5 prognostic markers (CDHR2, CEACAM7, CHST5, KRT7, and pcK1) as lactate-related signatures with a P value < 0.05 threshold. The coefficient of each marker was determined by Cox regression model, and the lactate score (risk score) was calculated as:

Risk score_i_ =


$$\sum_{j=1}^{n}Cj*\exp\text{i}j$$

based on the 5-gene lactate based signature, where “Risk score_i_” is the lactate score of the “i”-^th^ sample in the cohort, “*Cj*” is the regression coefficient of the j-^th^ marker in the model, and “exp *ij*” is expression of the j-^th^ markers in the i-^th^ sample.

The receiver operating characteristic (ROC) curve was used to evaluate the performance of the lactate score by calculating the area under curve (AUC). To validate the independent prognostic value of the lactate score, Kaplan-Meier survival analysis was performed in TCGA COAD and GEO CRC cohorts, where the cutoff value was set as the median lactate score. We also introduced an ROC curve to test its performance in TCGA COAD cohort.

### Predictive nomogram construction

To explore the prognostic effect of the lactate score, relevant clinical features, such as age, sex, AJCC tumor stage, and TNM stage, were included to perform a multivariate Cox regression analysis. Finally, we constructed a prognostic nomogram to predict the 1-, 3-, and 5-year OS of COAD patients in TCGA dataset. A calibration curve was constructed to evaluate the prediction accuracy of the nomogram. Alternatively, a decision curve analysis was used to determine the efficiency of our model in predicting the 1-, 3-, and 5-year survival.

### Application of lactate score in immunotherapy

Since immune checkpoints are vital for the immune response of tumors, we compared the expressions of these checkpoints between the high and low lactate score groups. Subsequently, the data of the urothelial carcinoma cohort treated with the PDL-1 inhibitor atezolizumab were subjected to the Kaplan-Meier survival analysis to validate the prognostic ability of the lactate score. We also performed a statistical analysis of the response rate of patients to the PDL-1 inhibitor.

### Somatic mutation and copy number variation

Among the somatic mutations derived from the WES of the COAD cohort, only single nucleotide variants (SNVs) and insertion-deletions (InDels) were included in the follow-up analysis, while silent mutations were ruled out. Both mutation frequency and SNV types have been reported. We also assessed the CNV for each gene and chromosome.

### Immune microenvironment evaluation

To detect immune cell infiltration in the COAD patients, we utilized the R package CIBERSORT (CIBERSORT, RRID : SCR_016955), which can estimate the abundances of 22 immune cells, based on gene expression data. We referred to immune-related signatures from previous studies and calculated their scores using gene set variation analysis (GSVA). Furthermore, we searched the MSigDB (V7.4) to identify chemokine-related pathways and obtained a series of chemokines. Subsequently, a correlation analysis was performed between lactate-related genes and immune cell infiltration. We compared the differences in immune cell infiltration, immune-related signatures, and chemokines among the lactate-related subtypes, while for the lactate phenotype-related subtypes, only immune cell infiltration was compared.

### Pathway enrichment analysis

Gene ontology (GO) and Kyoto Encyclopedia of Genes and Genomes (KEGG) pathway enrichment analyses were performed using the R package clusterProfiler (clusterProfiler, RRID : SCR_016884). The GO analysis included biological processes (BP), cellular components (CC), and molecular functions (MF). The GO terms and KEGG (KEGG, RRID : SCR_012773) pathways were considered to be significantly enriched for P values < 0.05. The hallmark pathway activity was analyzed using GSVA.

### Statistical analyses

Continuous variables were compared using Wilcoxon rank sum test between two groups and Kruskal-Wallis test among multiple groups. Categorical variables were analyzed using Fisher’s exact test. Kaplan-Meier survival was compared among subgroups using the log-rank test. The Cox proportional hazard model was applied to analyze the hazard ratio (HR) with a 95% confidence interval (95% CI). Both univariate and multivariate Cox regression analyses were used to evaluate survival. Correlation analysis was performed using Spearman or Pearson’s test. Unless otherwise stipulated, P < 0.05 was considered statistically significant. All statistical analyses were performed in R.

## Results

### Identification of lactate-related genes in colon adenocarcinoma

**Figure 1:**
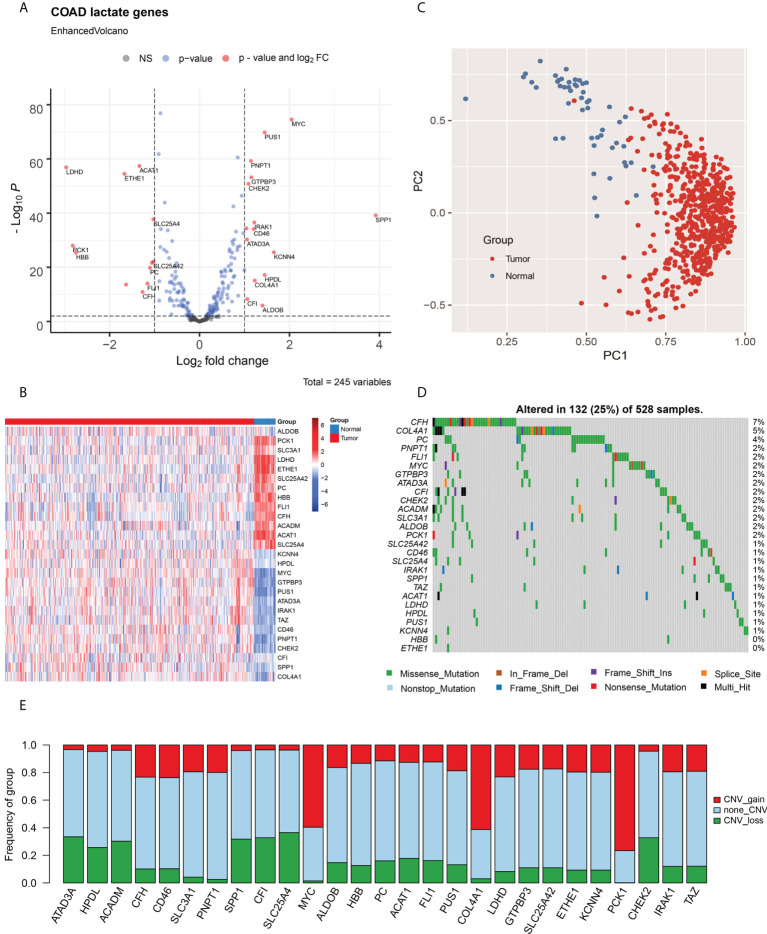
Identification of lactate-related genes in COAD and their genomic characteristics. **(A)** Volcano plot for analysis of differentially expressed genes in COAD; lactate-related genes with fold change > 2 and P < 0.01 are labeled in red. **(B)** Heat map of the lactate-related genes in COAD; the expression of genes are normalized. **(C)** The PCA, based on lactate-related genes, indicate that tumor samples and adjacent tissues are well-separated by first principal component (PC1) and second principal component (PC2). **(D)** Statistical analysis of mutational frequencies of lactate-related genes in COAD; only SNVs and InDels are included. **(E)** Statistical analysis of CNV types of lactate-related genes in COAD, including gain of CNV (red), loss of CNV (green), and no CNV (grey). NS, No Significant.

To explore the characteristics of lactate metabolism in COAD, 245 lactate genes from 13 lactate metabolic pathways were integrated in MSigDB. Based on the RNA-seq profile of TCGA COAD cohort, we identified 27 differentially expressed lactate genes (fold change > 2, FDR < 0.01) between the tumor samples and adjacent tissues. Among these, 15 genes are upregulated and 12 are downregulated in the tumors (**Figure 1A**). The expression levels of these lactate-related genes are elaborated in **Figure 1B**. Two lactate-related genes, namely secreted phosphoprotein 1 (*SPP1*) and *MYC* proto-oncogene (*MYC*), are upregulated by > 4-fold in the tumor samples; in fact, *SPP1* has the highest change, 16-fold. On the contrary, lactate dehydrogenase D (*LDHD*) has an extremely low expression in CRC patients, although its expression has been studied to a lesser extent than the other lactate-related genes. (**Figure 1A**; **Figure S1A**). Reportedly, *SPP1* plays an important role in mediating macrophage polarization and facilitating immune escape in lung cancer (24), and *MYC* is an oncogene encoding a transcription factor and participating in multiple metabolic pathways that induce cancer progression. Principal component analysis (PCA) results also indicate that the tumor samples and adjacent tissues are well-separated by the 27 lactate-related genes (**Figure 1C**).

The mutational landscape was generated using the WES dataset. Among all lactate-related genes, the complement factor H (*CFH*) is the most frequently mutated gene with a frequency of 7%, followed by collagen alpha-1 (IV) chain (*COL4A1*; 5%) and pyruvate carboxylase (*PC*; 4%) (**Figure 1D**). Majority of the SNVs are C > T transitions. (**Figures S1B, C**). Furthermore, we analyzed the CNV of lactate-related genes and discovered that *MYC* and *COL4A1*, both of which are upregulated in the tumors, mainly exhibit copy number amplification, thereby indicating a CNV-derived overexpression event in COAD (**Figure 1E**).

The level of immune cell infiltration in COAD patients was evaluated using CIBERSORT. Subsequently, we investigated the relationships between lactate-related genes and immune cell infiltration using Spearman’s correlation test (**Figure S2A**). Interestingly, we ascertained that *SPP1* is highly correlated to macrophage infiltration, which is consistent with its previously described role in mediating macrophage polarization and facilitating immune escape in lung cancer; hence, *SPP1* may have a similar function in COAD. We also discovered strong correlations among lactate-related genes, thereby indicating possible gene interactions in the lactate metabolism pathways (**Figure S2B**).

Thereafter, we used the Cox proportional hazard model to estimate the HR of lactate-related genes in both OS and PFS (**Table S3**). The results indicate that only *SPP1* (HR = 1.09, P = 0.033) is significantly associated with poor OS, hence making it a risk factor for COAD. On the contrary, acyl-coenzyme A dehydrogenase, C-4 to C-12 straight chain (*ACADM*; HR = 0.82, P = 0.044) and aldolase, fructose bisphosphate-B (*ALDOB*; HR = 0.91, P = 0.014) are considered as protective factors in OS. Generally, ALDOB is highly expressed in tumor samples; however, it appears as a protective factor in our analysis, signifying its role as a tumor suppressor in COAD. Moreover, for PFS, solute carrier family 25 member 42 (*SLC25A42*; HR = 1.30, P = 0.045), *SPP1* (HR = 1.08, P = 0.028), and *COL4A1* (HR = 1.21, P = 0.032) are risk factors, while phosphoenolpyruvate carboxykinase 1 (*PCK1*; HR = 0.88, P = 0.001) is a protective factor. We demonstrated that patients with downregulated *SLC25A42* (encoding ADP/ATP translocase) show poor PFS, indicating that dysfunction of ADP/ATP transformation may contribute to COAD progression.

### Lactate-related subtypes associated with prognosis and immune microenvironment

**Figure 2:**
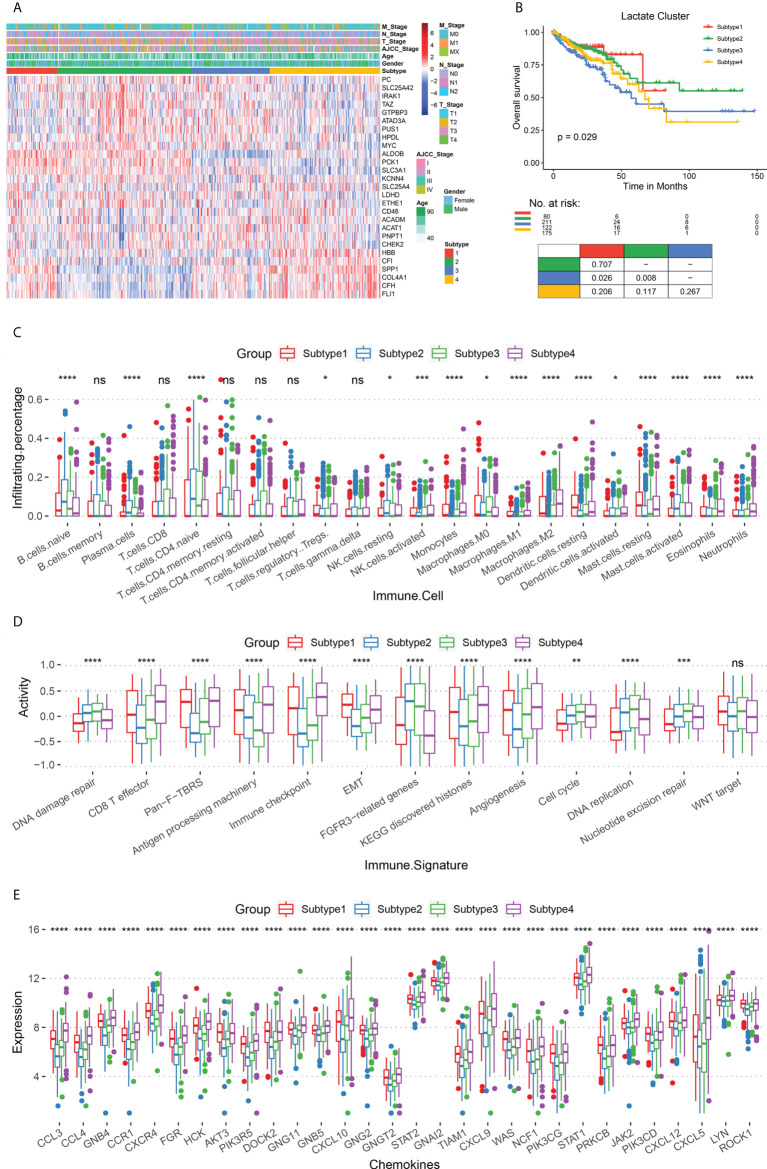
Comparison of survival rates and immune microenvironments of lactate-related subtypes. **(A)** Heat map of lactate-related genes in the four subtypes (subtypes 1 - 4), along with age, sex, AJCC tumor stage, and TNM stage. **(B)** The OS analysis among lactate subtypes; P values derived from a log-rank test. Pairwise comparison is shown at the bottom. **(C)** Box plot for comparing the infiltration percentage of 22 immune cells; immune cell infiltration has been evaluated by CIBERSORT. **(D)** Box plot for comparing the immune signatures, as calculated by GSVA. **(E)** Box plot for comparing the expression of chemokines. In **(C–E)**, * P < 0.05; ** P < 0.01; *** P < 0.001; **** P < 0.0001; ns, not significant (Kruskal-Wallis test).

To determine the association of lactate metabolism with immune microenvironment and prognosis of COAD patients, we divided the patients into different subtypes, based on their expressions of lactate-related genes. First, we performed consistent clustering analysis with subtypes 2 – 10 and evaluated the results using cumulative distribution function and incremental area analysis (**Figures S3A, B**). After comprehensive consideration of clustering effect and stability, we finally clustered the COAD patients into four lactate-related subtypes (subtypes 1 - 4) (**Figure 2A**; **Figure S3C**). Moreover, we compared the expressions of lactate-related genes among these subtypes, along with their age, sex, AJCC tumor stage, and TNM stage, and observed that each subtype has a high expression of specific lactate-related genes (**Figure 2A**). Additionally, there is no difference in age or sex among subtypes; however, subtype 2 includes more patients in the T1/2 stage, N1 stage, and AJCC stage I, as compared to the corresponding number of patients in the other subtypes (**Figure S4**). Furthermore, survival analysis demonstrated significant differences in OS (P = 0.029) and PFS (P = 0.038), and pairwise comparisons revealed that subtypes 1 and 2 have a significantly better OS than that of subtype 3 (P = 0.026 and P = 0.008, respectively) (**Figure 2B**). To better understand the changes in the biological processes among these subtypes, we evaluated the activities of the hallmark pathways using GSVA and compared the results using Kruskal-Wallis test. Incidentally, 50 pathways are significantly different (**Figure S5**). For instance, subtypes 1 and 4 have a higher expression of the interferon alpha response and epithelial-mesenchymal transition pathways than that in subtypes 2 and 3, while the G2M checkpoint and DNA repair pathways are upregulated in subtypes 2 and 3 (**Figure S5**).

Subsequently, we focused on analyzing the immune characteristics of these subtypes, such as immune cell infiltration, immune-related signatures, and chemokine secretion. Regarding immune cell infiltration, subtypes 2 and 3 have more naive B cells and T cells than subtypes 1 and 4, while subtypes 1 and 4 have more polarized macrophages than the other two (**Figure 2C**). With respect to immune-related signatures, initially, we obtained information from previous studies. Thereafter, our results confirmed that subtypes 1 and 4 are more active in epithelial-mesenchymal transformation, CD8+ T effector, and immune checkpoints than the other two subtypes, thereby suggesting a higher immune activity in them (**Figure 2D**). Subtypes 2 and 3 are upregulated in cell proliferation pathways, including DNA damage repair, DNA replication, and nucleotide excision repair. Finally, we analyzed the expression of chemokines in the four subtypes and found that subtypes 1 and 4 have a more severe inflammatory response than subtypes 2 and 3 (**Figure 2E**).

The immune infiltration and inflammatory response between subtypes 2 and 3 showed that a higher M2 macrophage infiltration in subtypes 3 patients with poorer survival (p ≤ 0.01, **Figure S6A**) than in subtypes 2. In addition, subtypes 3 patients showed higher angiogenesis (Angiogenesis, p ≤ 0.001), pan-fibroblast TGF-β(Pan-F-TBRs, p ≤ 0.0001), and epithelial interstitial transformation (EMT, p ≤ 0.05) (**Figure S6B**) compared with subtypes 2, suggesting that subtypes 3 patients had more robust pro-cancer activity. Also, subtype 3 patients had significantly higher expression levels for most chemokines (**Figure S6C**) than subtype 2, suggesting more vital white blood cell migration ability and pro-inflammatory response. In conclusion, we revealed an immunosuppressive microenvironment in subtypes 2 and 3, and an immune-activated microenvironment in subtypes 1 and 4, which, in turn, may contribute to different prognoses.

### Establishing lactate score based on lactate phenotype-related genes

**Figure 3:**
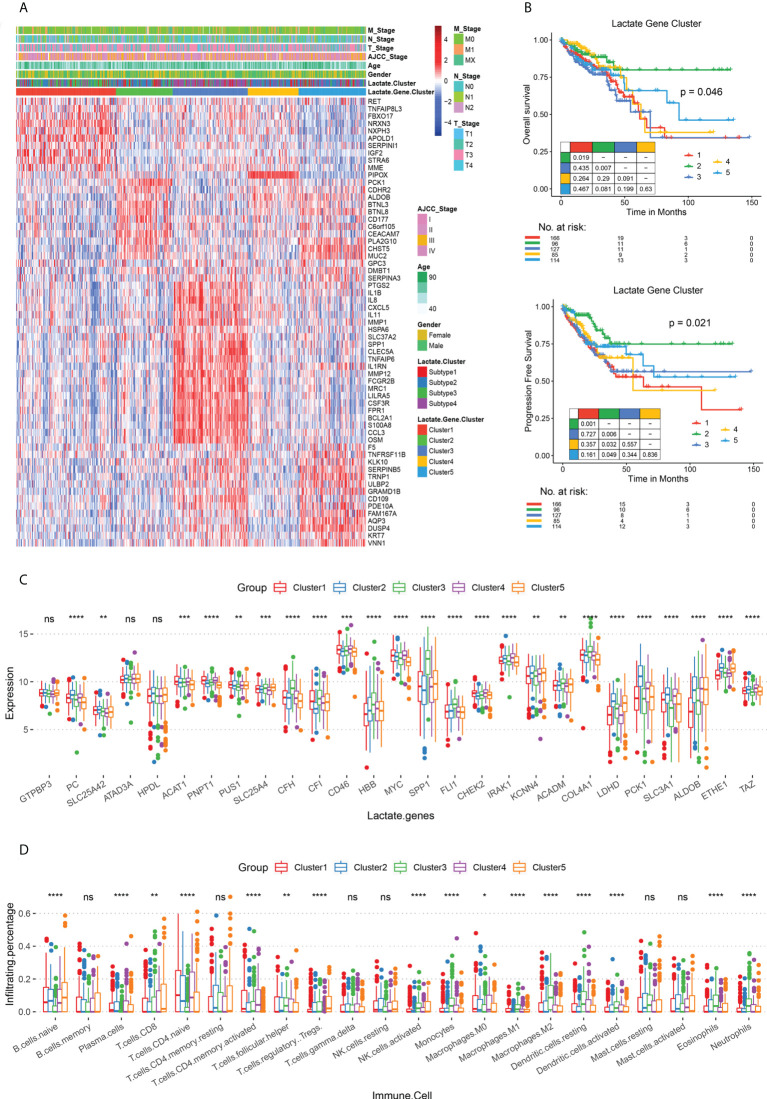
Lactate phenotype-related subtypes and differences in their survival rates and immune cell infiltration. **(A)** Heat map of lactate phenotype-related genes in five subtypes (clusters 1 - 5), along with age, sex, AJCC tumor stage, and TNM stage. The lactate-related subtypes are also labeled. **(B)** The OS and PFS analysis among subtypes; P values derived from a log-rank test. Pairwise comparison is also displayed. **(C)** Box plot for comparing the expressions of lactate-related genes among clusters 1 - 5. **(D)** Box plot for comparing the infiltration percentage of 22 immune cells; immune cell infiltration has been evaluated by CIBERSORT. In **(C, D)**, * P < 0.05; ** P < 0.01; *** P < 0.001; **** P < 0.0001; ns, not significant (Kruskal-Wallis test).

To explore the gene expression patterns of the four lactate subtypes, a series of subtype-specific DEGs were identified. A pairwise comparison of the subtypes has already established that subtypes 2 and 3 have the most significant difference in their survival statuses; hence, we took the intersection of the DEGs in these two subtypes. Consequently, 61 genes were extracted, and these were defined as lactate phenotype-related genes. Based on these genes, TCGA COAD patients were re-clustered into five lactate phenotype-related subtypes, namely clusters 1 - 5 (**Figure 3A**). Thereafter, the subtype-specific DEGs were identified in clusters 1 - 5, and biological function enrichment analyses, namely GO and KEGG pathway enrichment analyses, were performed. According to the GO analysis, the five subtypes show a significant difference in 250 BPs, 21 CCs, and 31 MFs (**Figures S7A–C**). Based on the KEGG pathway enrichment analysis, the DEGs are associated with 20 pathways (**Figure S7D**). Both OS (P = 0.046) and PFS (P = 0.021) are different among the five subtypes (**Figure 3B**). In fact, further comparisons revealed that cluster 2 has the best prognosis (both OS and PFS), while the survival rates of clusters 1 and 3 are the worst (**Figure 3B**). Interestingly, majority of the lactate-related genes are differentially expressed among the five clusters, except GTP-binding protein 3 (*GTPBP3*), ATPase family AAA domain containing 3A (*ATAD3A*), and 4-hydroxyphenylpyruvate dioxygenase like (*HPDL*) (**Figure 3C**). Moreover, immune cell infiltration levels are significantly different among the clusters (**Figure 3D**). Hence, we obtained this set of 61 lactate-phenotype genes that reflects the expression of the lactate-related genes.

**Figure 4:**
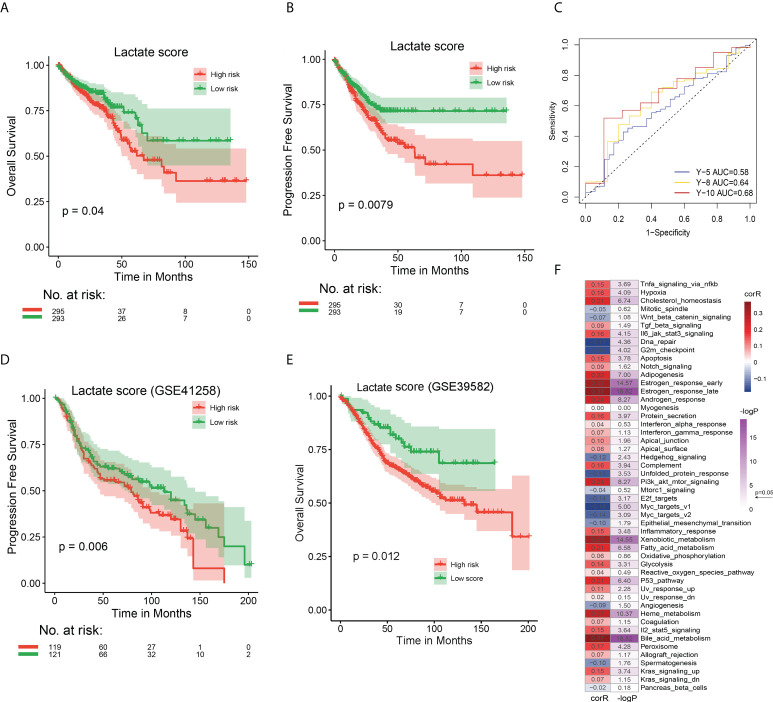
Prognostic performances of lactate score in TCGA COAD and GEO CRC cohorts and its association with hallmark pathways **(A)** The OS analysis of high and low risk groups in TCGA COAD cohort. **(B)** The PFS analysis of high and low risk groups in TCGA COAD cohort. **(C)** The ROC curve to evaluate the performance of lactate score by calculating the AUC. Prediction of 5-, 8-, and 10-year survival was performed. **(D)** The PFS analysis of high and low risk groups in GEO CRC cohort to validate the effect of lactate score. **(E)** The OS analysis of high and low risk groups in GEO CRC cohort to validate the effect of lactate score. **(F)** Correlation between lactate score and hallmark pathway activity. The activity of hallmark pathways was calculated by GSVA. The correlation coefficient and P value were evaluated by Spearman test. In **(A, B, D, E)**, P value has been derived from a log-rank test.

To build an effective evaluation system for prognosis, we further analyzed the 61 lactate-phenotype genes. Five prognostic markers, namely cadherin related family member 2 (*CDHR2*), CEA cell adhesion molecule 7 (*CEACAM7*), carbohydrate sulfotransferase 5 (*CHST5*), keratin 7 (*KRT7*), and *PCK1*, were screened by univariate Cox regression analysis. A Cox regression model was constructed to calculate the risk score (lactate score). There was no significant difference in the lactate score of the clinical phenotypes (**Figure S8**). Thereafter, we divided TCGA COAD patients into high- and low-risk groups using the median lactate score, and a survival analysis was performed to test the association between lactate score and survival status. As shown in **Figures 4A, B**, the high-risk group has a longer OS (P = 0.040) and PFS (P = 0.008) than that of the low-risk group. The prognostic efficacy of the lactate score was assessed using the ROC curve, which calculated the AUC, and our model exhibits an acceptable performance (Y-10 AUC = 0.68) (**Figure 4C**). Another CRC dataset (GSE41258) and colon cancer dataset (GSE39582) were used for validation of lactate scores, wherein patients with higher lactate scores had longer PFS (P = 0.006) and longer OS (P = 0.012) than the ones with low lactate scores; this is consistent with the results of TCGA COAD cohort (**Figures 4D, E**).

Subsequently, we compared the genomic characteristics of patients with different lactate scores, identified the preference for nonsynonymous mutations between patients with high and low lactate scores using Fisher’s exact test, and screened 731 differentially mutated genes. The 38 most frequently mutated genes are shown in **Figure S9A**. The phosphatidylinositol-4,5-bisphosphate 3-kinase, catalytic subunit alpha (*PIK3CA*) gene has the highest mutation frequency (28%) among all patients, and it occurs more the high lactate score group than in the low lactate score one (P = 0.032). Similarly, genes with > 10% mutation frequency are also more significantly observed in the high lactate score group than in the low lactate score one. Additionally, there are significant differences in the CNV between the two groups (**Figures S9B, C**). Hence, the lactate score is associated with the genomic characteristics of COAD patients, and patients with high lactate scores have complicated mutations and CNV events.

We attempted to determine the relationship between lactate score-based groups and the different subtypes, namely lactate-related subtypes (subtypes 1 - 4), lactate phenotype-related subtypes (clusters 1 - 5), and immune subtypes (C 1 - 6). A Sankey diagram was used to visualize the patient distribution. Incidentally, patients with high lactate scores were highly likely to be located in cluster 1 and subtype 3 (**Figure S10A**). However, further comparisons revealed that patients in cluster 2 and subtype 1 have the highest lactate scores (**Figures S10B, C**). We also calculated the correlation between lactate score and activities of the hallmark pathways using Spearman test and discovered that the lactate score is significantly correlated with majority of the pathways, especially estrogen response, xenobiotic metabolism, and bile acid metabolism (**Figure 4F**). In fact, a comparison between high- and low lactate score patients revealed that 32 of 50 pathways are significantly different (**Figure S11**). In summary, lactate score is an effective signature to distinguish the different states of COAD patients.

**Figure 5:**
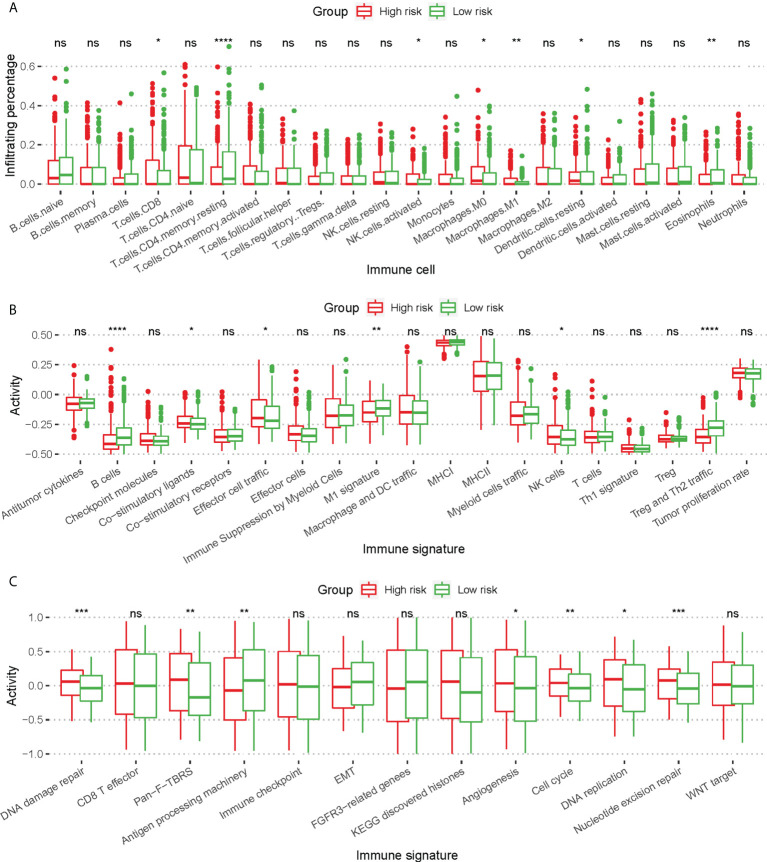
Difference of immune cell infiltration and immune signature activity between high risk patients and low risk patients. **(A)** Box plot for comparing the infiltration percentage of 22 immune cells; immune cell infiltration has been evaluated by CIBERSORT. **(B, C)** Box plot for comparing the activity of immune signature. In **(A–C)**, * P < 0.05; ** P < 0.01; *** P < 0.001; **** P < 0.0001; ns, not significant (Kruskal-Wallis test).

The infiltration of 22 immune cells indicated that low risk patients showed significantly higher infiltration of T cells CD4 memory resting (p ≤ 0.0001) and eosinophils (P ≤ 0.01), as well as higher M0/M1 macrophage infiltration (p ≤ 0.05, **Figure 5A**). Low risk patients showed a significantly higher B cell (p ≤ 0.0001) activity and Th2 traffic, p ≤ 0.0001) activity (**Figure 5B**). In addition, we found significant differences in Co-stimulatory ligands, Effector cell traffic, M1 signature, and NK cell activity between 2 groups of patients (**Figure 5B**). Lastly, by analyzing the immune signature activity, low risk patients showed significantly higher antigen processing machinery activity (p ≤ 0.01) and lower DNA damage repair, and nucleotide excision repair activity (p ≤ 0.001, **Figure 5C**), indicating a more robust immune response and lower genomic instability.

### Lactate score as a signature for prognosis and immunotherapy

**Figure 6:**
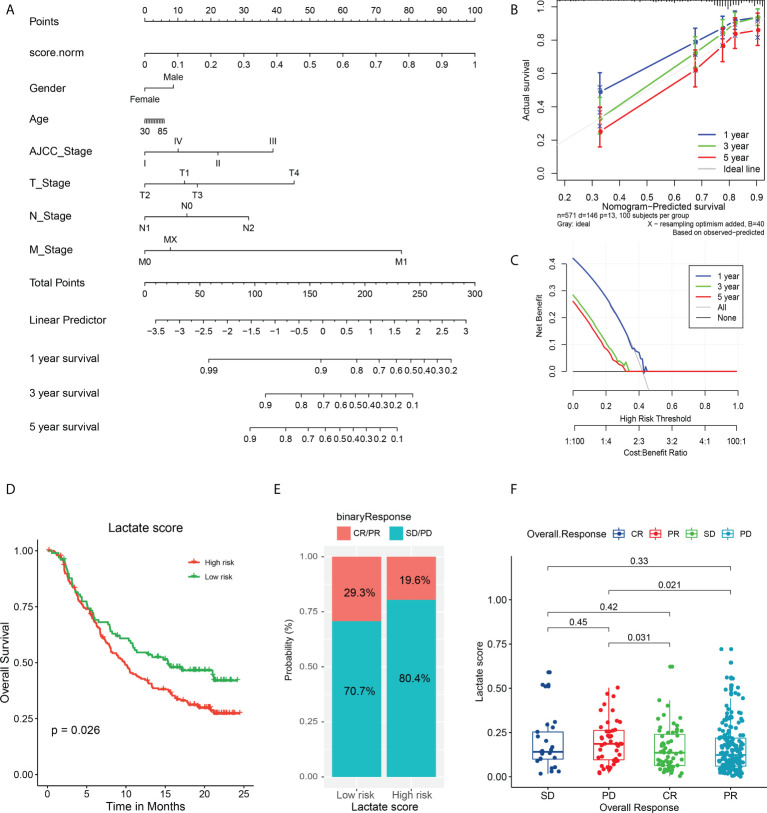
Validation of nomogram in predicting survival and application of lactate score in prognosis of immunotherapy. **(A)** A prognostic nomogram predicting 1-, 3-, and 5-year OS of TCGA COAD cohort. **(B)** The calibration curve of nomogram to predict 1-, 3-, and 5-year OS. **(C)** The decision curve analysis used to compare the efficiency of nomogram to predict 1-, 3-, and 5-year OS. **(D)** The OS analysis of high and low risk groups in urothelial carcinoma cohort treated with PDL-1 inhibitor atezolizumab; P value has been derived from a log-rank test. **(E)** The response of immunotherapy in groups with high or low lactate score. The response status includes PR, CR, stable disease (SD), and PD. high lactate score patients: CR/PR (29/99); SD/PD (70/99), low lactate score patients: CR/PR (39/199); SD/PD (160/199) **(F)** Comparison of lactate score among different response statuses; P value has been derived from Wilcoxon rank sum test.

To further explore the effect of lactate score on clinical prognosis, multivariate Cox regression analysis was performed by incorporating clinical features for correction. This demonstrated that lactate score is an independent prognostic factor in COAD that influences the survival of patients (**Figure S12**). Moreover, to support the clinical analysis, we combined the lactate score and clinical features and created a nomogram (**Figure 6A**). A calibration curve was designed to evaluate the prediction accuracy of 1-, 3-, and 5-year survival rates. Interestingly, the predicted risk is highly similar to the real risk, and this confirms the reliability of our nomogram (**Figure 6B**). Decision curve analysis was also used to determine the efficiency of our model in predicting 1-, 3-, and 5-year survival rates, and the model was found to be most efficient in predicting the 1-year survival (**Figure 6C**).

Furthermore, we attempted to determine if the lactate score can serve as a biomarker for predicting the response to immunotherapy. First, we analyzed the association between the expression of immune checkpoints and lactate score. Interestingly, CD27 and CCR4 expressions are significantly high in low lactate score patients, while the others do not exhibit any significant differences; this suggests that the low lactate score subset of patients is more sensitive to immune checkpoint inhibitors than the others are (**Figure S13**). Additionally, a cohort of urothelial carcinoma patients treated with PDL-1 inhibitor atezolizumab was studied for validation, and it revealed that patients with higher lactate scores have poorer prognoses than the ones with lower lactate scores (P = 0.026) (**Figure 6D**). Moreover, the high lactate score group exhibited a lower response to atezolizumab with only 19.6% of the patients expressing a partial response (PR) or complete response (CR), as compared to the responses of the low lactate score group (29.3%) (**Figure 6E**). Further analysis revealed that the patients who experienced progressive disease (PD) post-treatment had higher lactate scores than those who had exhibited PR (P = 0.021) or CR (P = 0.031) (**Figure 6F**). Therefore, lactate score has the ability to reflect the status of immune checkpoints, to a certain extent, which, in turn, can serve as an evaluation factor for prognosis of immunotherapy.

## Discussion

This is the first study to construct a lactate score module for evaluating the efficacy of immunotherapy in CRC. The lactate microenvironment in CRC patients is extraordinarily complex due to the presence of lactate derived from both tumor and intestinal flora. Tumor-derived lactate is a by-product of aerobic glycolysis in tumor cells, and its continuous accumulation causes acidification of the TME. Considering the significant role of lactate in the TME, we investigated and identified 27 lactate-related genes in COAD. Among them, *SPP1* plays a crucial role in metastasis of multiple cancers by inducing the degradation of extracellular matrix (ECM) *via* the matrix metalloproteinase (MMP) family (25). This study demonstrated that the increased expression of *SPP1* worsens the prognosis of CRC patients by mediating tumor evasion from immune responses. Inversely, the impact of copy number loss of SPP1, which is a frequent occurrence, is yet to be elucidated. The *MYC* oncogene can alter the glucose metabolism in tumors by attenuating E2F1 expression (26). Reportedly, LDHD is the only member of the LDH family that can utilize D-lactate (27). In clear cell renal cell carcinoma, low expression of *LDHD* is a promising predictor of poor prognosis; however, its role in CRC has not been previously reported. Hence, we may be the first to report that a decreased expression of *LDHD* in CRC patients possibly leads to lactate accumulation and causes immune cell infiltration and PD-L1 inhibition. Incidentally, *PCK1* is reported as an oncogene in CRC (28). In this study, in spite of being copy-duplicated, it appeared to be a protector with a low expression. According to a previous study, CRC patients who did not undergo preoperative radiotherapy exhibited high plasma HBB levels (29). The plasma HBB may be secreted by tumor cells, which is consistent with it low expression observed in tumor cells in this study. Moreover, we demonstrated that these lactate genes are closely associated with immune infiltration and can be used to estimate the prognosis of CRC patients.

Tumor-infiltrating immune cells (TICs) can cause immunosuppression and immune evasion, and the different immune cells play different roles in the TME. Tumor-promoting TICs dominate during tumorigenesis and tumor progression, and they prevent tumor-suppressive TICs from functioning. Incidentally, both tumor-promoting and tumor-suppressing TICs can be targeted during immunotherapy. To investigate the correlation between the lactate-related genes and immune microenvironment, we clustered the CRC patients into four subtypes. Survival analysis revealed that subtypes 1 and 2 have a significantly better OS than that of subtype 3; in fact, the latter does not exhibit any direct connection between lactate level and survival. Subtypes 1 and 4 may be defined as immunologically hot, i.e., they can benefit more from immunotherapy than subtypes 2 and 3, which correspond to immunologically cold. The survival of subtype 3 is significantly worse than subtype 2 may be caused by the difference in M2 macrophages infiltrates (p ≤ 0.01, **Figure S6A**). Studies have shown that macrophages with local infiltration can undergo M2 type polarization in the tumor microenvironment, thereby exerting immunosuppressive effects and promoting intratumoral angiogenesis, tumor proliferation, invasion, and invasion metastasis (30, 31).In addition, subtypes 3 patients showed higher angiogenesis (Angiogenesis, p ≤ 0.001), pan-fibroblast TGF-β(Pan-F-TBRs, p ≤ 0.0001), and epithelial interstitial transformation (EMT, p ≤ 0.05) (**Figure S6B**) compared with subtypes 2, suggesting that subtypes 3 patients had more robust pro-cancer activity. which is consistent with a worse prognosis for patients (**Figure 2B**). Studies have shown that chemokine-oriented white blood cell migration in cancer is associated with exacerbation, leading to disease progression with immune or inflammatory components (32, 33). Also, subtype 3 patients had significantly higher expression levels for most chemokines (**Figure S6C**) than subtype 2, suggesting more vital white blood cell migration ability and pro-inflammatory response.

five lactate phenotype-related subtypes were clustered, based on 61 lactate phenotype-related genes that were identified from subtypes 1 – 4. The five clusters exhibit significant differences in the expression of lactate genes, such as *SPP1*, *MYC*, *LDHD*, and *PCK1*. Moreover, cluster 2 has the highest lactate score and the best prognosis (both OS and PFS). It also has more CD8+ T cell infiltration than the other clusters do, and hence, it corresponds to immunologically hot. These results suggest that high lactate levels in CRC patients may induce complex tumor immune infiltration, thereby attenuating the tumor’s response to immunotherapy, such as PD-L1 blockade. We further verified this hypothesis in a clinical trial of urothelial cancer patients treated with the PD-L1 blocker atezolizumab. The study revealed that lactate score is an independent prognostic factor of CRC, which can be used as a clinical guide for predicting the progression and as an evaluation factor for the efficacy of immunotherapy.

Tumor mutation burden (TMB) is a clinically related parameter that highlights the molecular characteristics associated with immunotherapy responses (34). Consequently, an elaborate TMB analysis to detect the underlying mutation subsets responsible for immunogenicity may lead to substantial optimization of biomarker accuracy and improve therapeutic targeting of neoantigens (35). Incidentally, *PIK3CA* exhibits a high mutation frequency in high-risk CRC patients. In fact, a recent report indicated that *PIK3CA* mutations promote glycolysis and proliferation by inducing the β-catenin/SIRT3 axis in cervical cancer (36). Therefore, *PIK3CA* mutation is a potential mechanism of lactate accumulation in CRC patients, and it can be utilized as a therapeutic target. Since we could not obtain sufficient CRC immunotherapy datasets for evaluation of immunotherapy effects, we used a urothelial cancer dataset. Therefore, this study can partially illustrate the effects of lactate on immunotherapy in CRC.

## Conclusions

In conclusion, since lactate levels influence tumor immune infiltration and tumor mutation burden, the lactate score can be used to estimate prognosis in CRC patients. Additionally, it can be used as a clinical guide for predicting the progression of CRC patients and as an evaluation factor for the effect of immunotherapy.

## Data availability statement

Publicly available datasets were analyzed in this study. This data can be found here: https://www.cbioportal.org/study/summary?id=coadread_tcga_pan_can_atlas_2018, https://www.ncbi.nlm.nih.gov/geo/query/acc.cgi?acc=GSE41258, https://ega-archive.org/studies/EGAS00001002556.

## Author contributions

DZ: Investigation, data curation, formal analysis, writing-original draft. YJ: Investigation, data curation, writing-original draft, writing-review and editing. HC: Methodology, formal analysis. JY: Investigation, data curation, formal analysis. YS: Data curation, formal analysis. HF: Data curation, formal analysis. XY: Data curation, formal analysis. XS: Funding acquisition, writing–review and editing. MS: Data curation, formal analysis, investigation, writing–review and editing, supervision, funding acquisition. All authors contributed to the article and approved the submitted version.

## Funding

This work was supported by the grants from Natural Science Foundation of Guangdong Province of China (2020A1515010603), Guangzhou Science and Technology Project (201904010405), Administration of Traditional Chinese Medicine of Guangdong Province of China Project (No. 20211356, 20221261).

## Conflict of interest

The authors declare that the research was conducted in the absence of any commercial or financial relationships that could be construed as a potential conflict of interest.

## Publisher’s note

All claims expressed in this article are solely those of the authors and do not necessarily represent those of their affiliated organizations, or those of the publisher, the editors and the reviewers. Any product that may be evaluated in this article, or claim that may be made by its manufacturer, is not guaranteed or endorsed by the publisher.
