# Supplementary material for: Lactate: A regulator of immune microenvironment and a clinical prognosis indicator in colorectal cancer
Source: Front Immunol. 2022 Aug 26;13:876195. doi: 10.3389/fimmu.2022.876195 (PMC9458902; doi:10.3389/fimmu.2022.876195)
Supplement: Supplementary file 1 [file DataSheet_1.docx]

Lactate: a regulator of immune microenvironment and a clinical prognosis indicator in colorectal cancer

Appendix A. Supplementary material

Supplementary figures
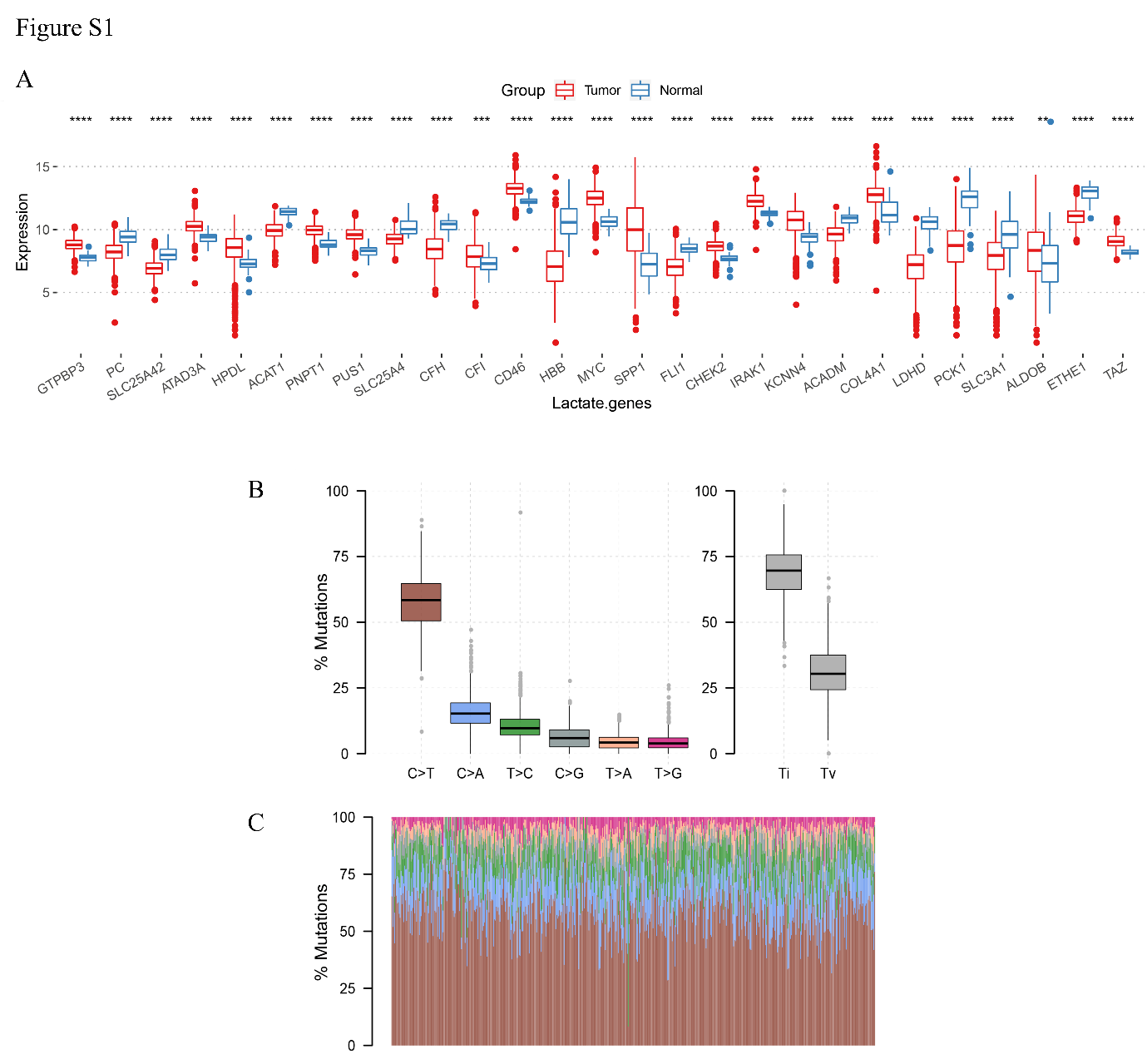


Figure S1. Expression of lactate-related genes between tumor samples and adjacent tissues and statistical analysis of SNVs

A. Box plot for comparing the expression of lactate-related genes between tumor samples and adjacent tissues.

B. Percentage of SNV types; Ti are purine to purine or pyrimidine to pyrimidine transitions, and Tv are purine to pyrimidine or pyrimidine to purine transversions.

C. Statistical analysis of SNV types in all TCGA COAD patients.


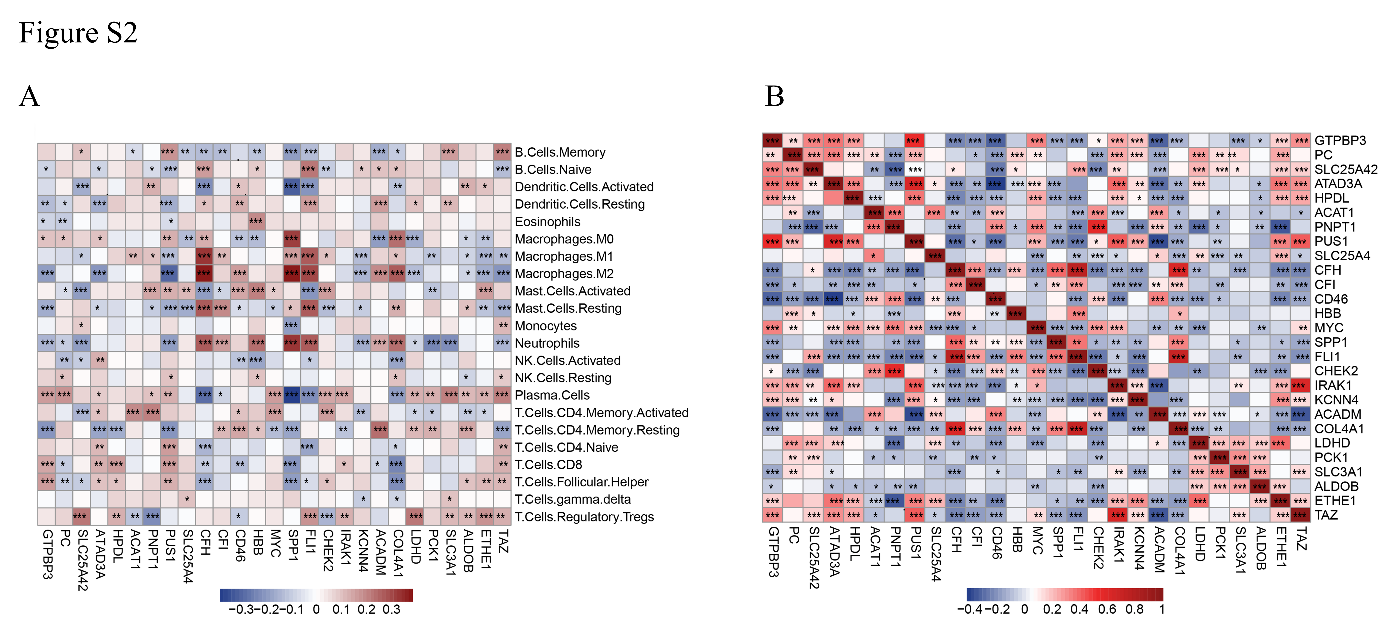
Figure S2. Correlation of lactate-related genes and immune cell infiltration and correlation among genes

A. Correlation of lactate-related genes and immune cell infiltration. The correlation coefficient and P value have been evaluated by Spearman test.

B. Correlation of lactate-related genes and immune cell infiltration. The correlation coefficient and P value have been evaluated by Pearson test.


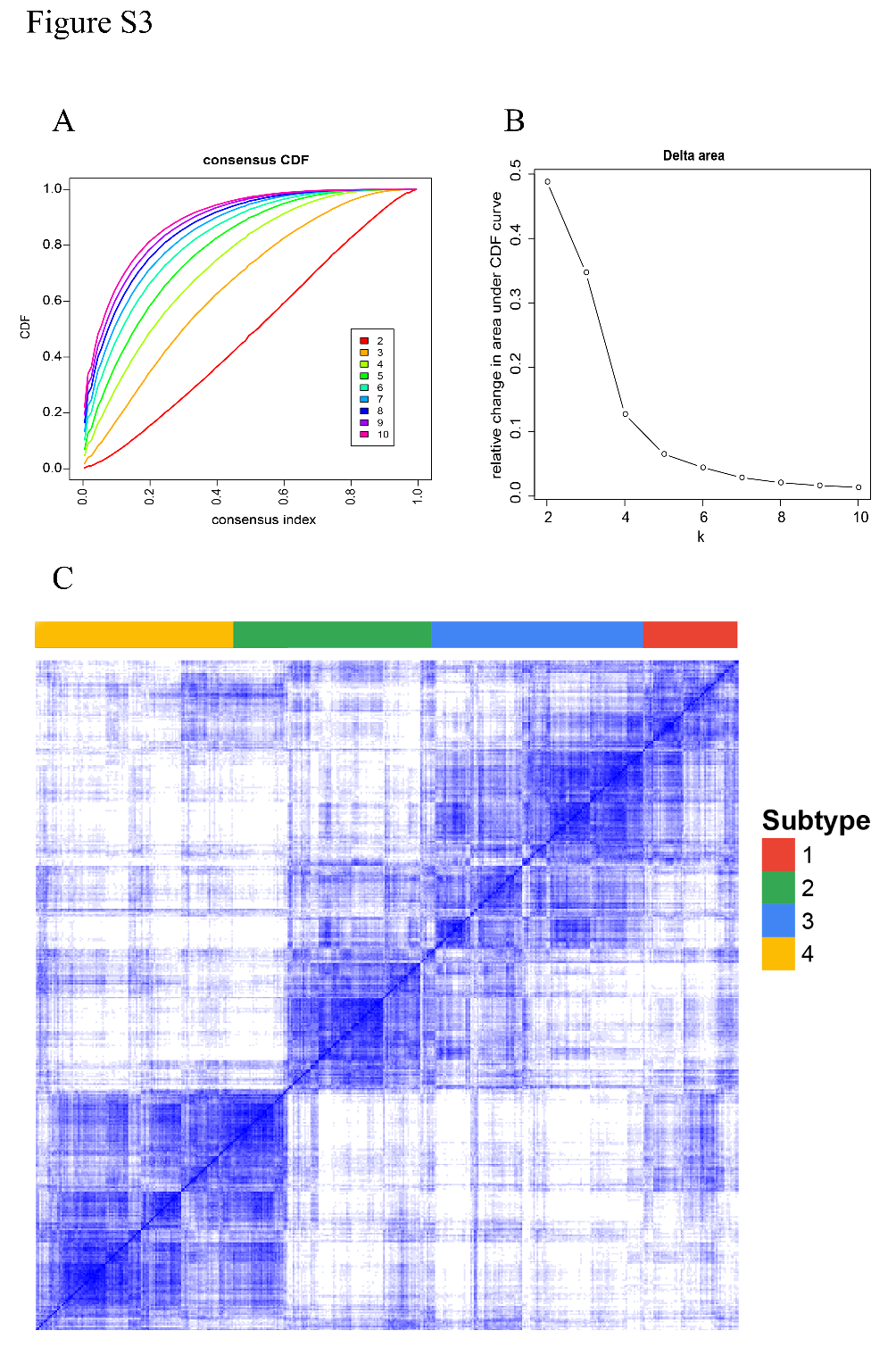
Figure S3. Consistent clustering analysis based on lactate-related genes

A. The curves of cumulative distribution function (CDF) of consistent clustering analysis; the number of clusters range from 2 to 10.

B. Relative changes of the area under CDF curves. There is a considerable decline from clusters 4 to 5 and no significant decline from clusters 5 to 6.

C. Heat map for distance of samples with a consistent clustering analysis of four clusters. The distance ranges from 0 (white) to 1 (blue).


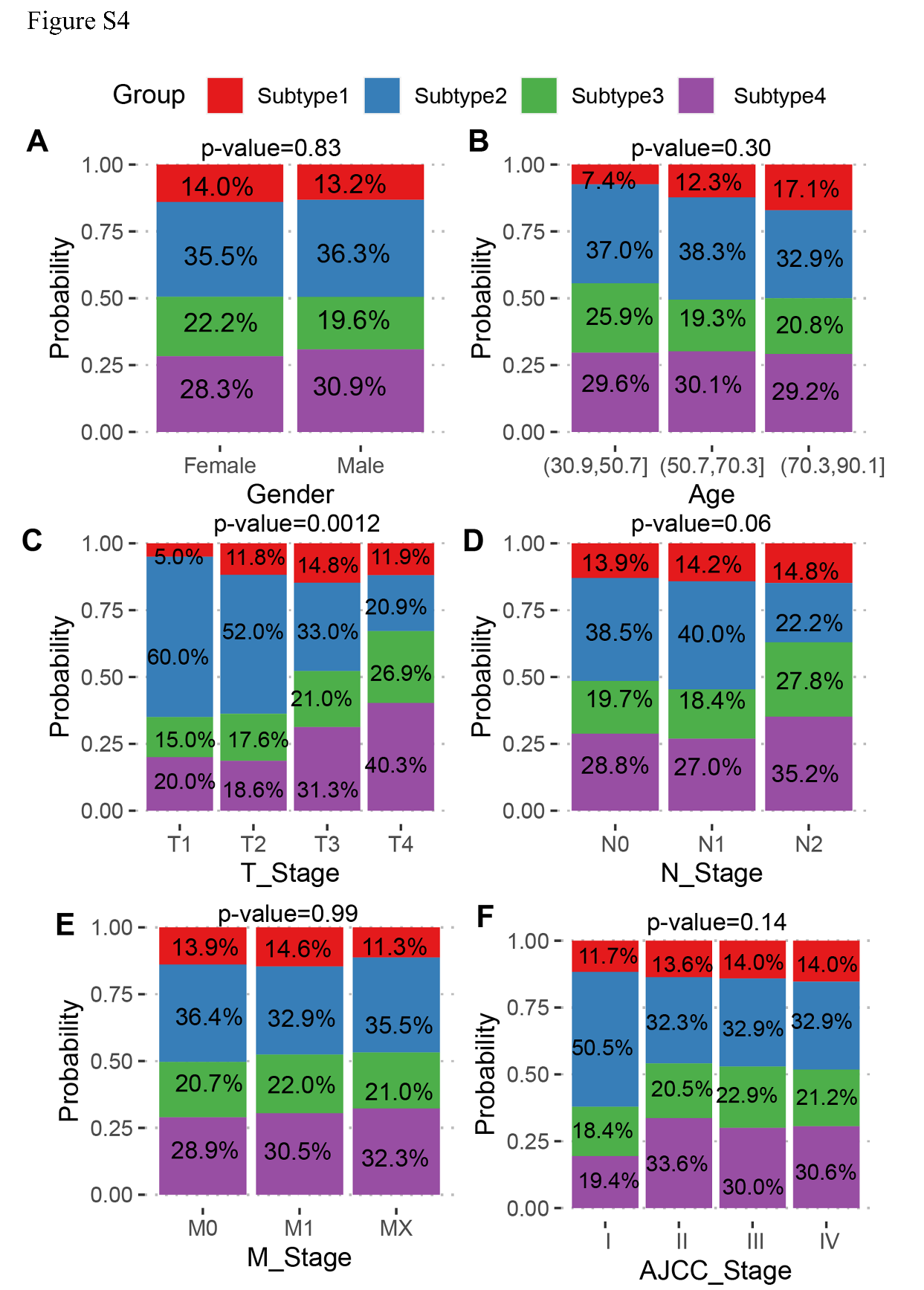
Figure S4. Distribution of clinical phenotypes in lactate-related subtypes

A. Distribution of sex (female and male) in lactate-related subtypes.

B. Distribution of age (30.9 – 50.7, 50.7 – 70.3, and 70.3 – 90.1) in lactate-related subtypes.

C. Distribution of T1-4 stage in lactate-related subtypes.

D. Distribution of N1-3 stage in lactate-related subtypes.

E. Distribution of M1-3 stage in lactate-related subtypes.

F. Distribution of AJCC stages I - IV in lactate-related subtypes.


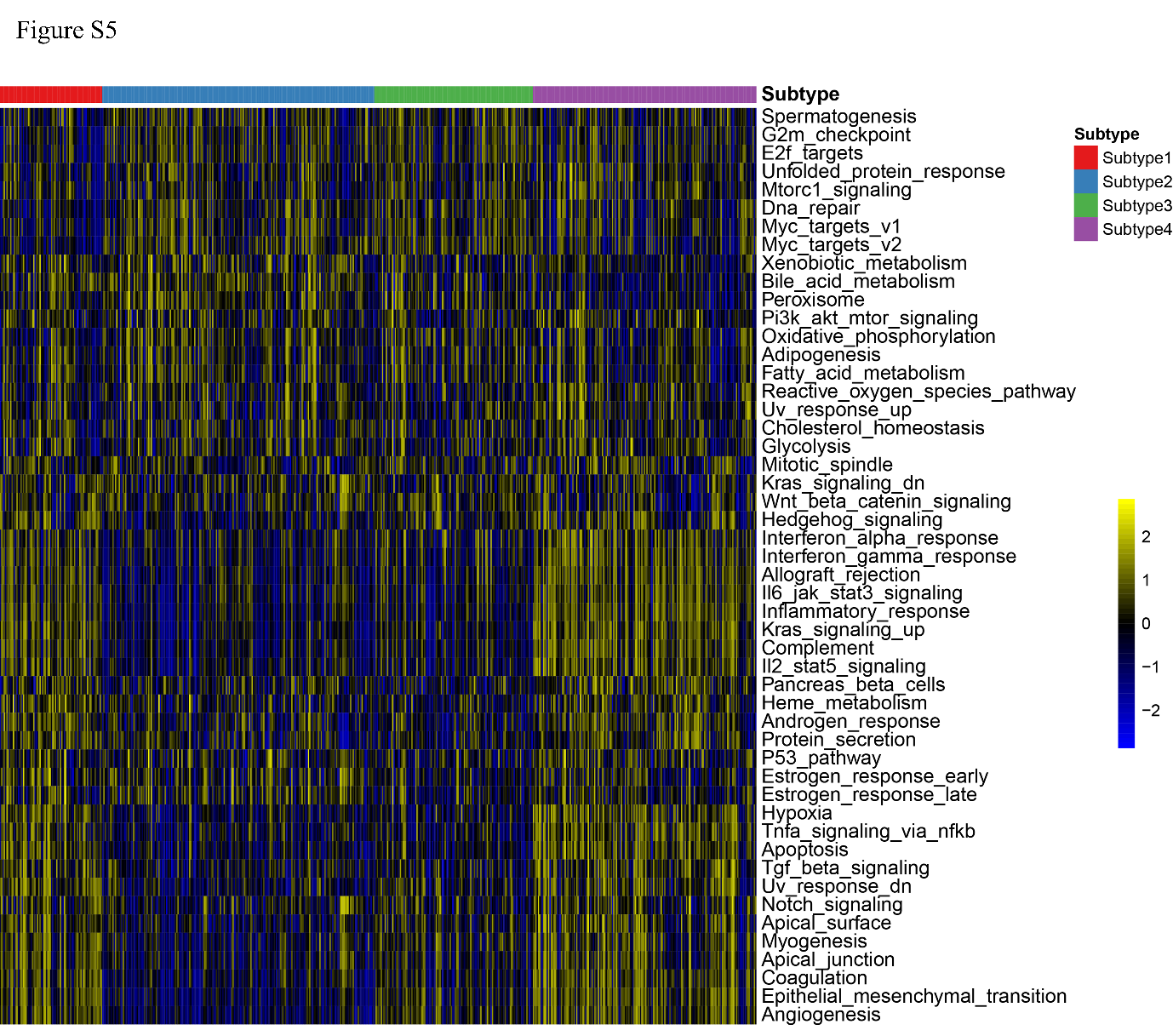
Figure S5. Hallmark pathways with different activities in lactate-related subtypes

Heat map showing the activity of 50 hallmark pathways, as calculated using GSVA in lactate-related subtypes. The pathways are defined as significantly different if the Kruskal-Wallis test P value < 0.05.


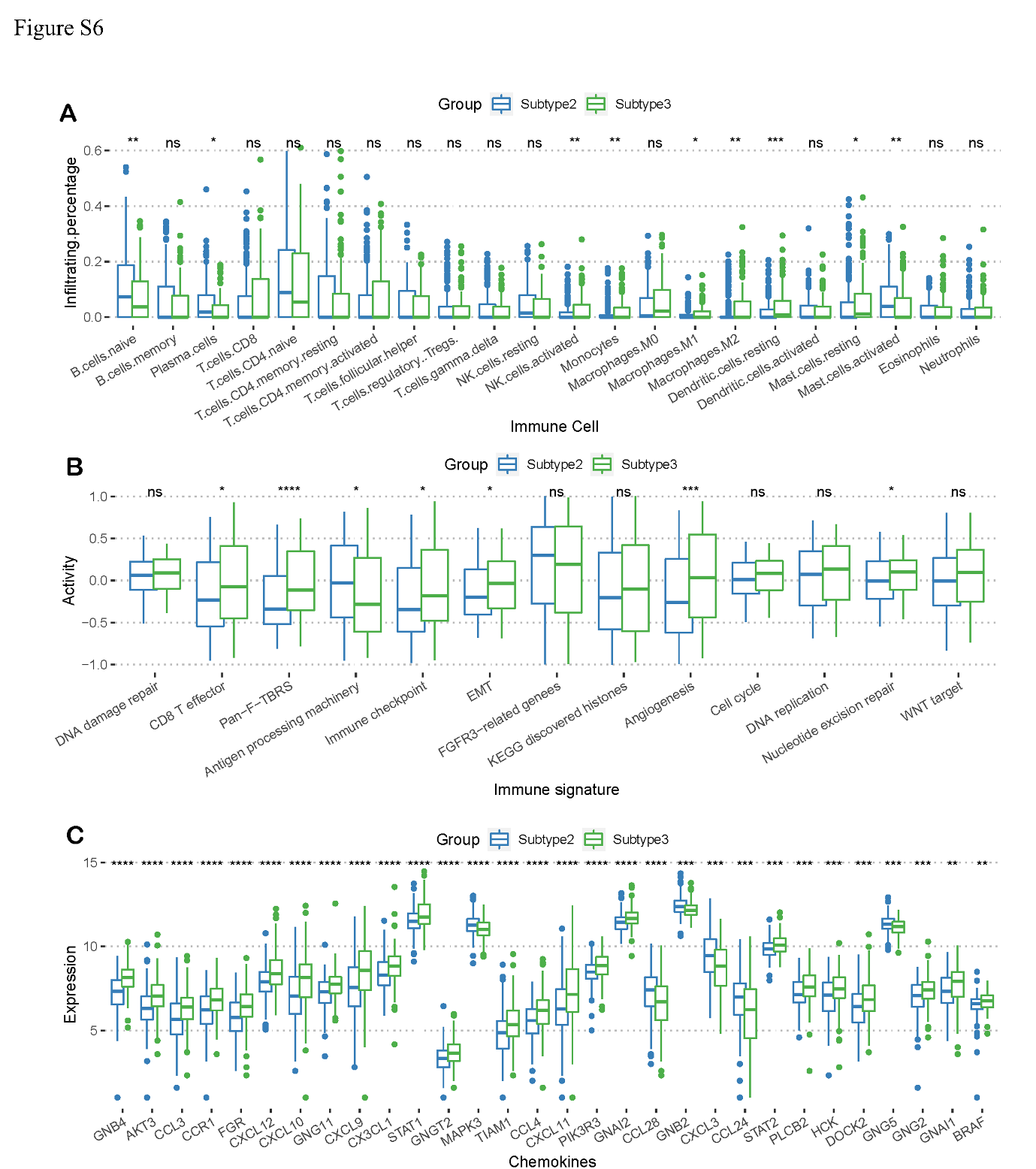


Fig S6. Comparing the immune cell infiltration, immune signature activity and chemokines expression between subtypes 2 and 3.

1. Box plot for comparing the infiltration percentage of 22 immune cells; immune cell infiltration has been evaluated by CIBERSORT.
2. Box plot for comparing the activity of immune signature.
3. Box plot for comparing the expression of chemokines.

In (A-C), * P < 0.05; ** P < 0.01; *** P < 0.001; **** P < 0.0001; ns, not significant (Kruskal-Wallis test).


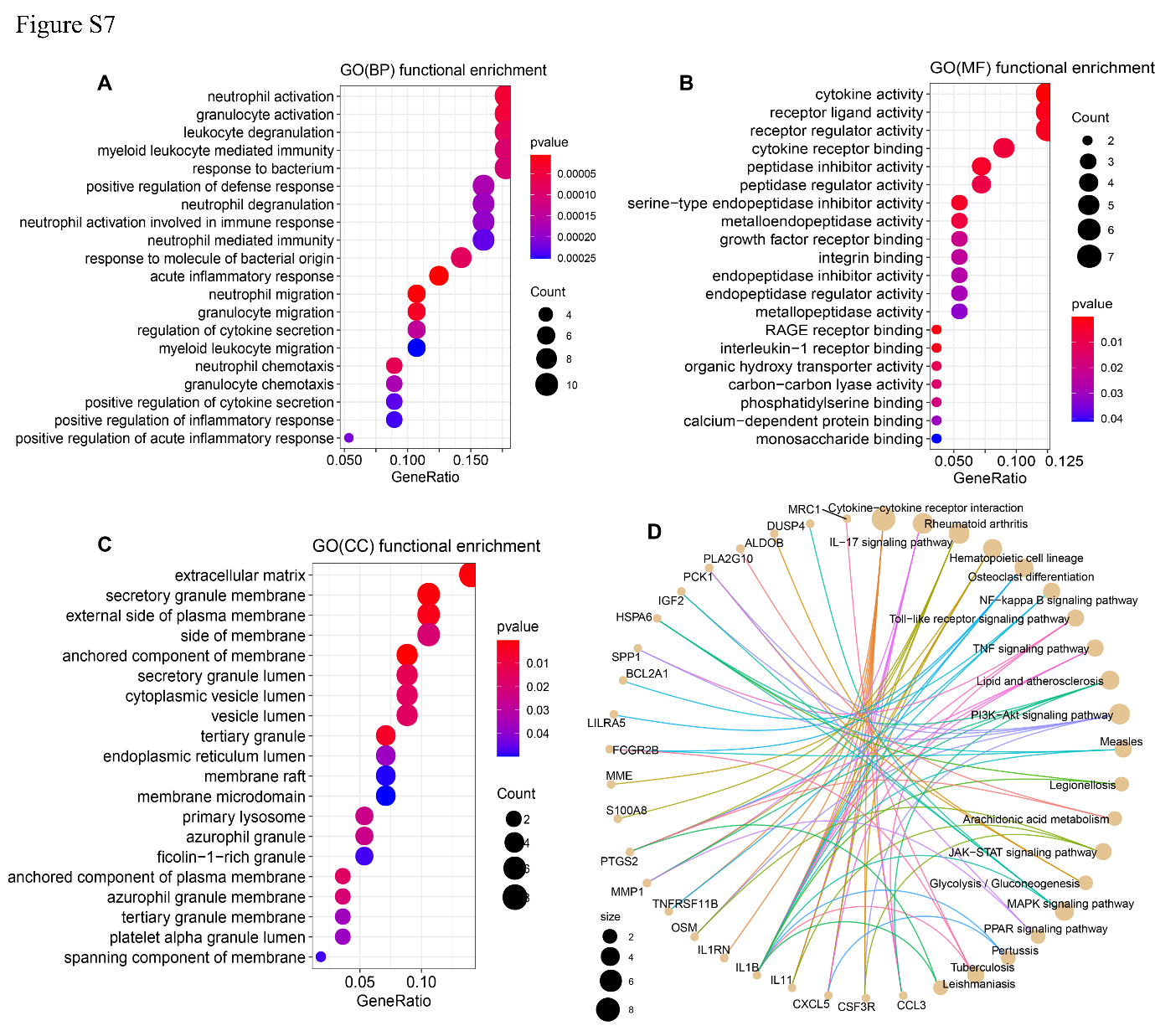
Figure S7. Pathway enrichment analysis based on subtype-specific DEGs in lactate phenotype-related subtypes

A - C. The result of GO analysis. The top 20 enriched BPs (A), CCs (B), and MFs (C) are displayed. The x-axis represents the proportion of genes in the pathway. The size of the points represents the number of genes. The P value is represented by color.

D. A network for genes and KEGG pathway interactions. Point means a gene or a pathway, and gene in the pathway is linked by a line. The size of the points represents the number of genes in the pathways.


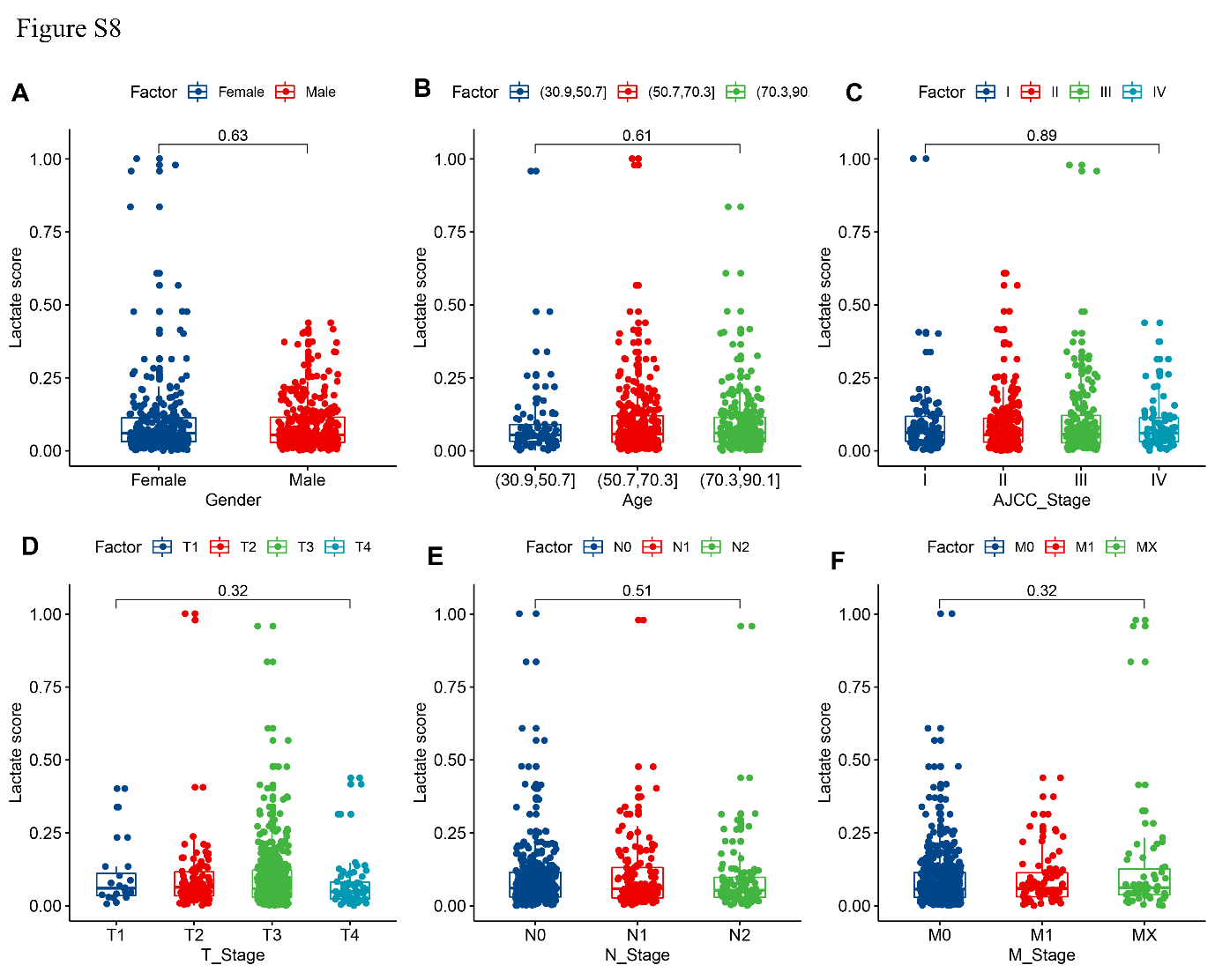
Figure S8. Comparison of lactate score in clinical phenotypes

A - F. Box plot displays the lactate score in sex (A), age (B), T-stage (C), N-stage (D), M-stage (E), and AJCC tumor stage (F). There is no significant difference (Kruskal-Wallis test).


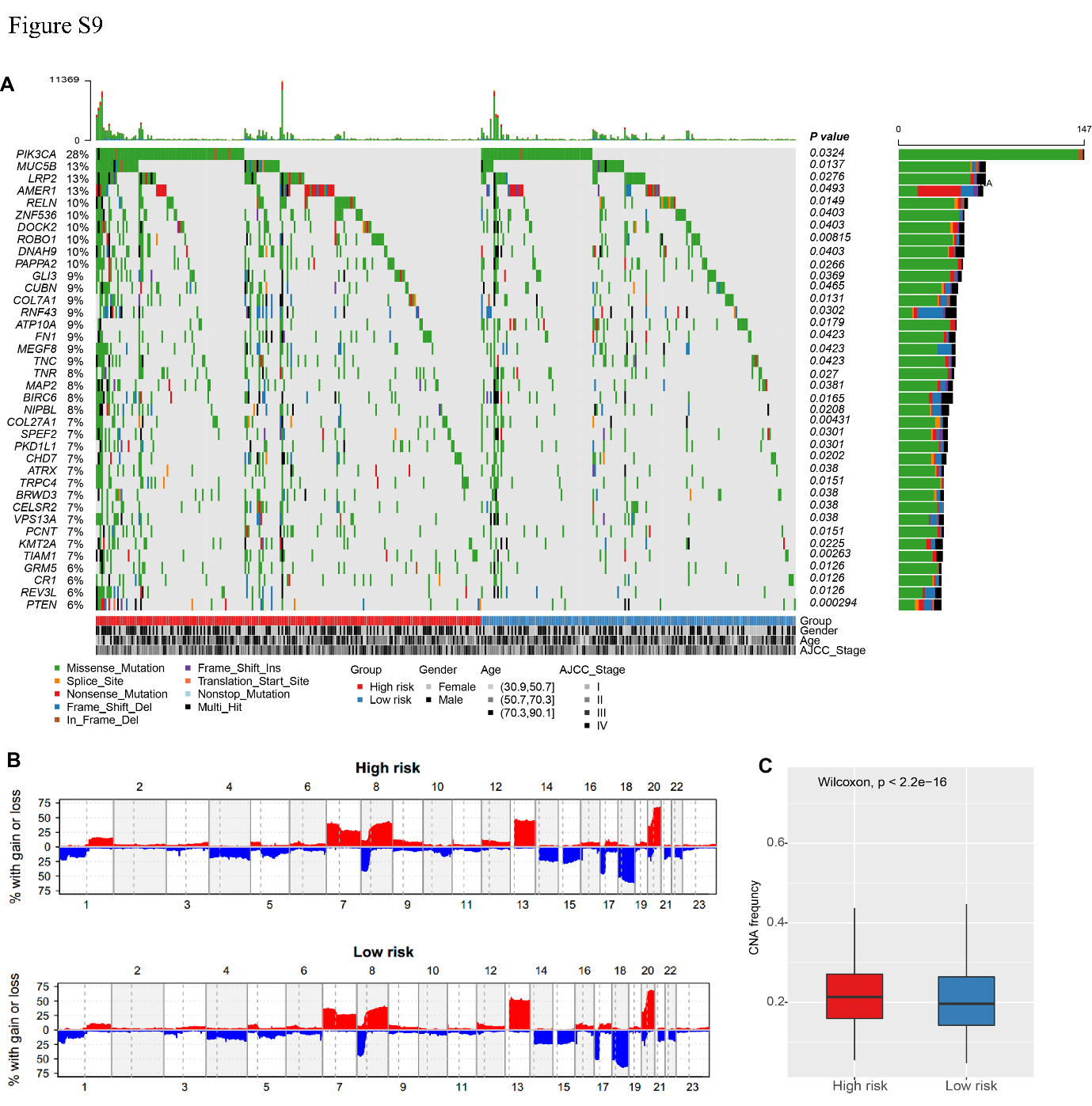
Figure S9. Somatic mutation and CNV in high and low risk groups

A. Top 38 genes with significantly different mutation frequency between high and low risk groups; only SNVs and InDels are included. Comparison has been performed by Fisher’s exact test, and P value < 0.05 is significant.

B. Distribution of CNVs in chromosomes of high and low risk groups.

C. Box plot compares the CNV frequency between high and low risk groups; P value has been derived from Wilcoxon rank sum test.


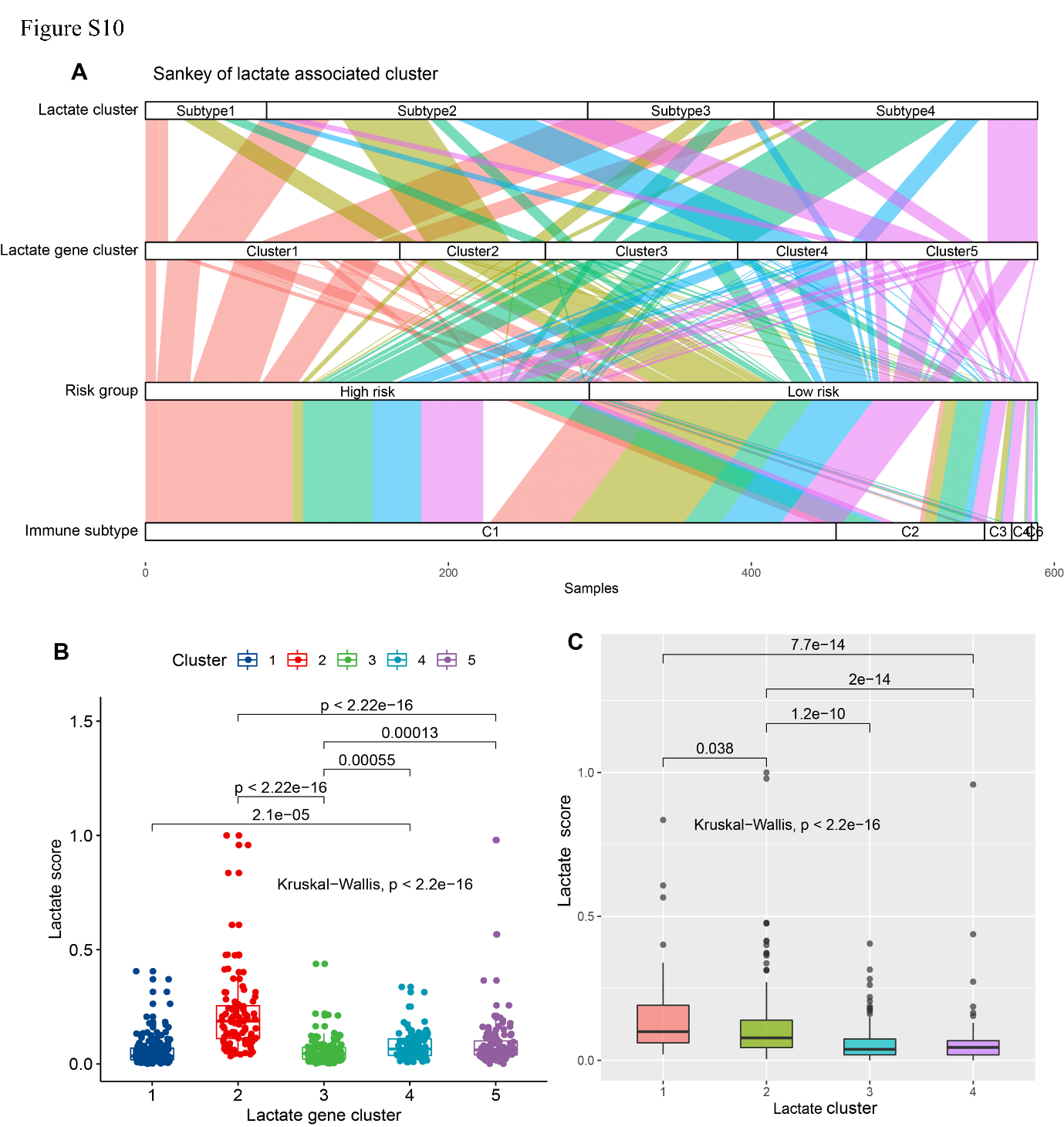
Figure S10. Comparing the lactate score among different subtypes

A. Sankey diagram to visualize the distribution of patients in lactate score-based groups (high risk and low risk), lactate-related subtypes (subtypes 1 - 4), lactate phenotype-related subtypes (clusters 1 - 5), and immune subtypes (C 1 - 6).

B - C. The lactate score is further compared among lactate-related subtypes (subtypes 1 - 4) (B) and lactate phenotype-related subtypes (clusters 1 - 5) (C). Kruskal-Wallis test has been used in multi-group comparisons, and Wilcoxon rank sum test has been used for pairwise comparisons.


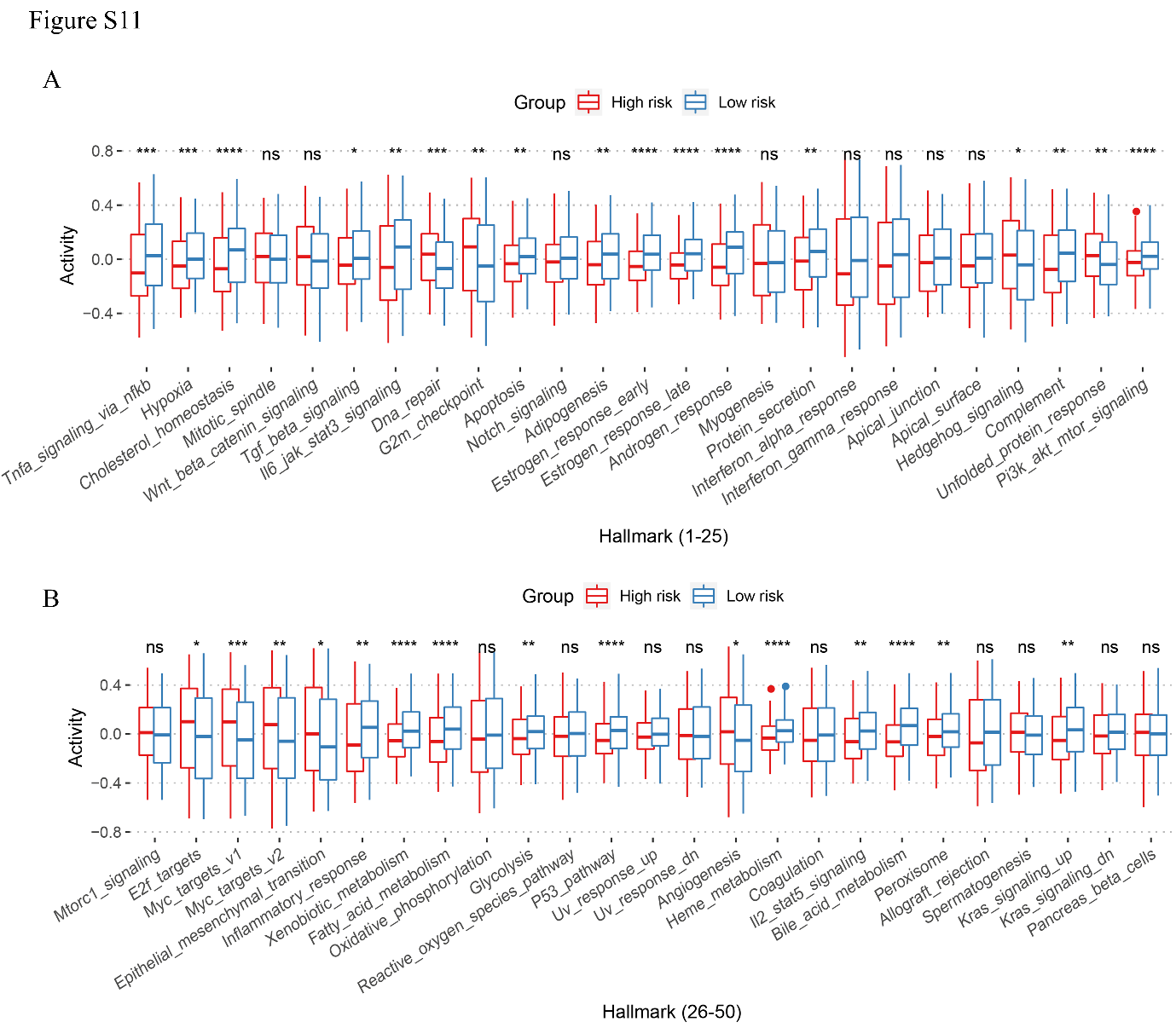
Figure S11. Comparing the hallmark pathway activity between high and low risk groups

A - B. The activity of hallmark pathways has been compared between high and low risk groups, and the results are displayed in A (1 - 25) and B (26 - 50). * P < 0.05; ** P < 0.01; *** P < 0.001; **** P < 0.0001; ns, not significant (Wilcoxon rank sum test).


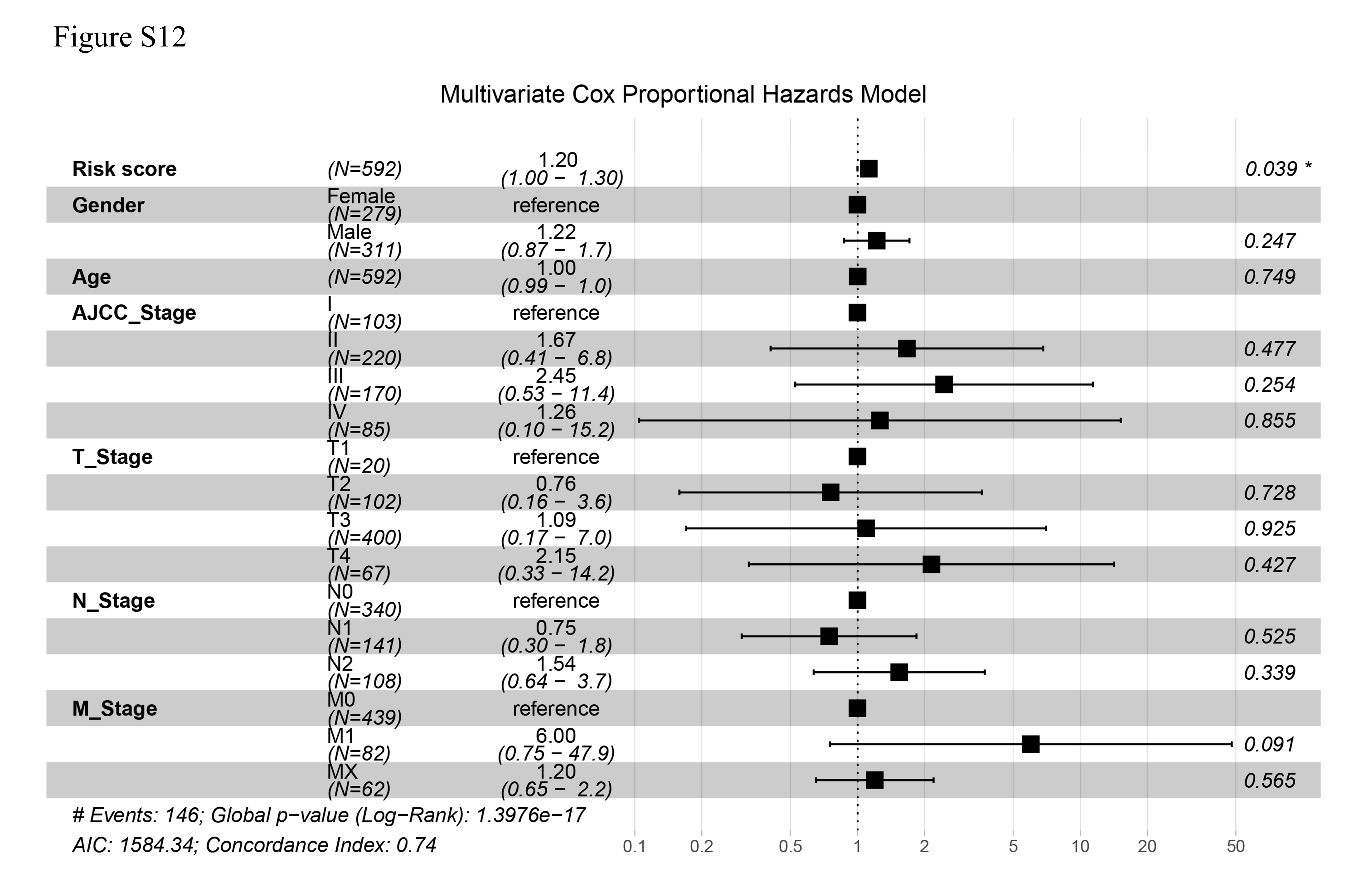
Figure S12. The forest plot of multivariate Cox proportional hazard model for lactate score and clinical variables. * P < 0.05.


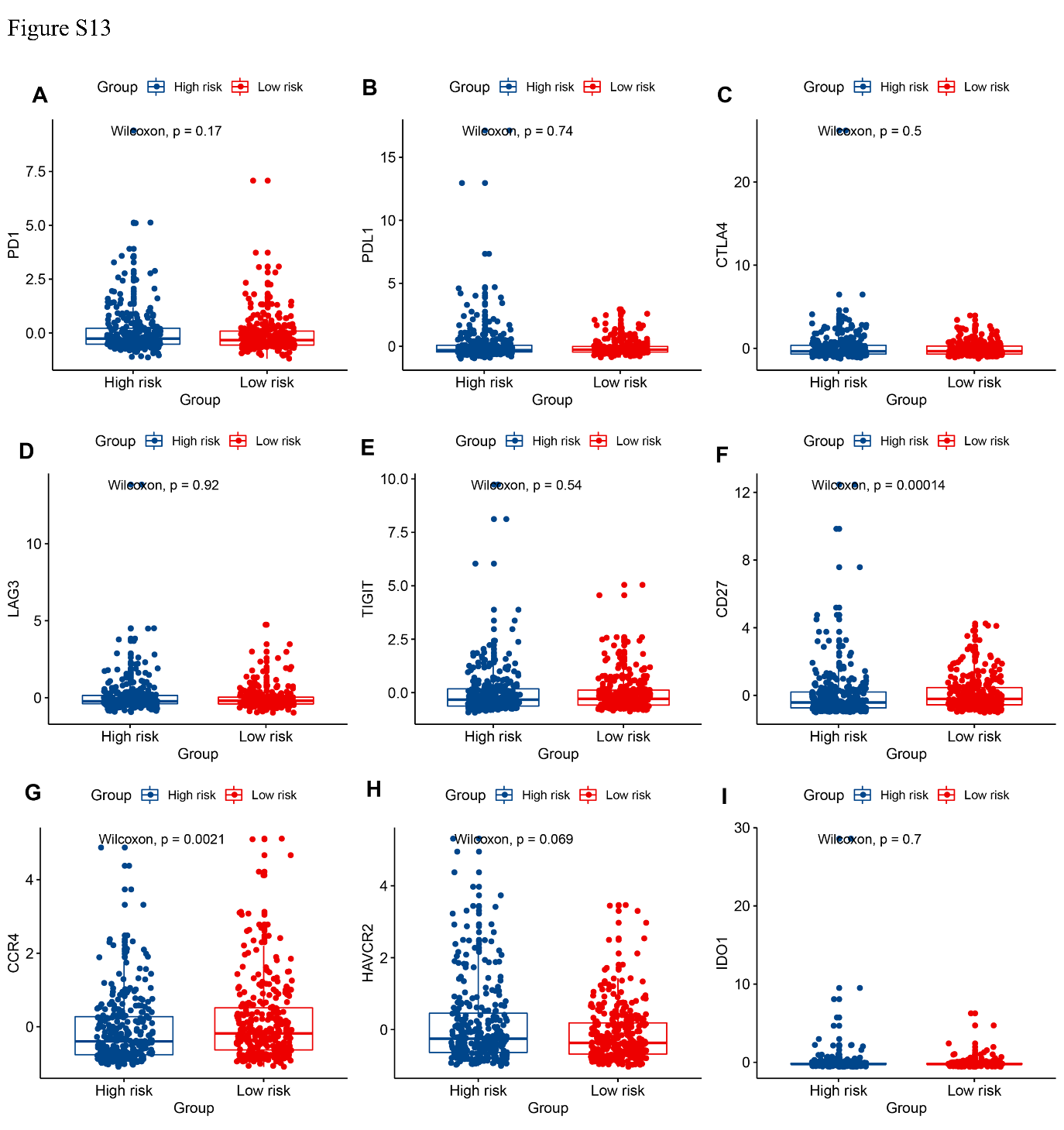
Figure S13. The expression of immune checkpoints in high and low risk groups

A - I. Box plot shows the expression immune checkpoints, including programmed cell death protein 1 (PD1) (A), PD-L1 (B), cytotoxic T-lymphocyte-associated protein 4 (CTLA4) (C), lymphocyte-activation gene 3 (LAG3) (D), T cell immunoreceptor with Ig and ITIM domains (TIGIT) (E), CD27 (F), CCR4 (G), hepatitis A virus cellular receptor 2 (HAVCR2) (H), and indoleamine-pyrrole 2,3-dioxygenase (IDO1) (I). P value has been derived from Wilcoxon rank sum test.

Supplementary Tables

Table S1. Description of clinical characteristics for COAD cohort in TCGA

Table S2. Lactate related pathways and genes involved in each pathway

| Pathways | Genes |
| --- | --- |
| HP INCREASED SERUM LACTATE | MT-ND4, NDUFA4, TUFM, NDUFA13, SURF1, COX6A2, COX4I1, COX14, LONP1, SYNJ1, UQCRC2, LYRM7, NDUFB9, TMEM70, BCS1L, TXN2, CARS2, NAXE, MT-ND1, MT-TF, HIBCH, TK2, NDUFA10, MRPS22, SLC25A10, OCRL, GTPBP3, MRPS34, PET100, LIAS, PMPCB, AIFM1, PNPO, PC, COQ2, SCO2, WARS2, SDHA, MT-CO1, NDUFA12, TRMT5, DARS2, GFM2, RNASEH1, PNPLA8, TRMT10C, NDUFA9, MT-TV, MT-TS2, TANGO2, NDUFB8, AARS2, TWNK, PDHA1, FDX2, SFXN4, PDSS2, MT-ND5, POLG2, LIPT2, TIMM22, PET117, COX5A, POLG, GFM1, TIMM50, MTO1, LIPT1, MT-TK, TMEM126B, MT-ND6, NDUFAF6, MT-TN, ECHS1, GOT2, ISCA1, CHCHD10, SLC25A42, ACAD9, MT-TL1, MT-ATP6, MICOS13, UQCRQ, NDUFS2, ADAMTS13, NDUFS8, MT-TH, NDUFS1, NDUFA1, CYC1, NARS2, SCO1, ATAD3A, MRPS28, HPDL, MRPL44, NDUFV1, MTFMT, COX10, LDHA, ACAT1, SLC7A7, COX6B1, OGDH, PYGL, EARS2, SLC25A3, HSD17B10, MRPS14, ATPAF2, NDUFAF1, FOXRED1, RRM2B, C1QBP, SOD1, C12orf65, GYS2, MIPEP, MRPS16, MT-TL2, NDUFAF3, PNPT1, MDH2, SERAC1, NDUFS3, MT-TI, MT-CO3, DGUOK, PUS1, MT-ND2, MT-TQ, TACO1, FBXL4, TARS2, MPC1, MRPL3, FASTKD2, YARS2, UQCC3, RARS1, COX15, DNM1L, COX20, NDUFV2, NDUFA11, MT-TW, NDUFS7, HTRA2, COQ8A, PDHX, ISCU, SLC19A3, SLC25A26, RARS2, COX8A, HMGCL, AGK, PDSS1, FARS2, COQ4, CLPB, NDUFAF2, DNAJC19, NDUFAF5, NGLY1, MRPL12, HS6ST2, COQ9, ACAT2, RMND1, TRMU, DLD, MT-ND3, NDUFS4, LRPPRC, CA5A, MECP2, UQCRB, TSFM, MT-CO2, NDUFA2, MT-TP, SLC25A4, PDP1 |
| HP INCREASED CSF LACTATE | NDUFA11, MT-ND4, NDUFS4, NDUFA4, COX6B1, SURF1, NDUFA13, NDUFAF1, COX4I1, COX14, LONP1, HSD17B10, TIMMDC1, ECHS1, TXN2, NDUFV1, FOXRED1, NDUFS6, NAXE, MT-ND1, MT-TF, NDUFA10, C12orf65, TMEM126B, SLC39A8, NDUFAF3, PNPT1, MDH2, NDUFS1, MRPS34, NDUFS3, NDUFS8, MT-CO3, PET100, MTFMT, NDUFAF6, AIFM1, MT-ND2, PDHA1, MT-TQ, SCO2, NDUFB10, SDHA, TACO1, MT-CO1, NDUFA12, DARS2, GFM2, TRMT10C, NUBPL, MT-TV, MT-TS2, NDUFB8, COX15, COX20, NDUFV2, NDUFB9, MT-ND5, MT-TW, NDUFS7, SLC13A3, SLC25A19, PET117, NDUFAF4, HTRA2, COQ8A, PDHX, GFM1, SLC19A3, NDUFAF2, RARS2, COX8A, MT-ND6, MT-TN, MPV17, NDUFS2, NDUFA1, MT-ND3, NDUFAF5, MT-TL1, MT-ATP6, NDUFAF8, MT-TH, NDUFB11, NDUFB3, RMND1, NARS2, SUCLG1, LIPT1, NDUFA6, LRPPRC, HPDL, NDUFA9, MECP2, MT-TK, TRAPPC12, COA8, MT-CO2, NDUFA2, COX10 |
| HP ABNORMAL BRAIN LACTATE: LEVEL BY MRS | NDUFAF3, MT-ND5, SURF1, MT-TW, MT-CO3, MT-TL1, C12orf65, MT-TQ, NDUFS2, MT-TH, HSD17B10, MT-ND4, NDUFB8, SUCLG1, MT-CO1, MT-ND6, COG8, HPDL, MT-ND1, MT-TS2, MT-TF, MT-CO2, SCO2, DNM1L |
| HP INCREASED LACTATE DEHYDROGENASE LEVEL | TP53, CFH, ACAD9, SLC7A7, PLEC, LYST, CFI, TET2, CD46, SLC4A1, HBB, RHAG, MYC, PIEZO1, MVK, SPP1, MPL, PLA2G6, FLI1, SLC25A13, STAT4, CHEK2, CALR, RB1, IRAK1, PNPLA2, VPS13A, RPS14, USB1, HELLPAR, JAK2, KCNN4, GAA |
| HP ABNORMAL LACTATE DEHYDROGENASE LEVEL | TP53, CFH, SLC7A7, FKTN, PLEC, LYST, TET2, CD46, SLC4A1, POMK, SPP1, SIL1, PLA2G6, FLI1, ACADM, POMGNT1, RB1, USB1, LARGE1, GAA, COL4A1, RXYLT1, POMT2, ACAD9, GATA1, CFI, HBB, RHAG, MYC, PIEZO1, MVK, DAG1, FKRP, MPL, SLC25A13, STAT4, CHEK2, CALR, IRAK1, POMGNT2, PNPLA2, VPS13A, INPP5K, RPS14, POMT1, HELLPAR, CRPPA, JAK2, KCNN4, B4GAT1, B3GALNT2 |
| GOBP LACTATE METABOLIC PROCESS | PARK7, LDHD, PNKD, HAGH, HIF1A, LDHA, LDHC, MIR210, PFKFB2, TIGAR, MRS2, TP53, SLC25A12, PER2, ACTN3 |
| GOBP LACTATE TRANSMEMBRANE TRANSPORT | EMB, SLC5A12, SLC5A8, SLC16A8, SLC16A1, SLC16A3, SLC16A7 |
| GOMF LACTATE DEHYDROGENASE ACTIVITY | LDHAL6A, LDHD, LDHA, LDHB, LDHC, LDHAL6B |
| GOMF L LACTATE DEHYDROGENASE ACTIVITY | LDHAL6A, LDHA, LDHB, LDHC, LDHAL6B |
| GOMF LACTATE TRANSMEMBRANE TRANSPORTER ACTIVITY | SLC5A12, SLC5A8, SLC16A8, SLC16A1, SLC16A3, SLC16A7 |
| HP LACTIC ACIDOSIS | NDUFA11, SLC37A4, NDUFS4, ATP5F1E, PDHX, TUFM, LYRM4, PCK1, MT-TS2, NDUFB3, LONP1, MT-CO3, LYRM7, TMEM70, LARS1, BCS1L, TIMMDC1, NFU1, NDUFV1, HSD17B10, NDUFS6, NAXE, MT-ND1, TKFC, PREPL, NDUFS2, COA6, MT-TF, GTPBP3, MRPS34, FOXRED1, PDHB, LIAS, PC, NDUFV2, COQ2, SCO2, SLC25A13, PPM1B, NDUFB10, WARS2, SDHA, USP18, MT-CO1, MT-ND2, SLC25A19, PNPLA8, TRMT10C, MRPS7, TANGO2, PHKA2, AARS2, SLC3A1, SFXN4, NDUFA10, TYMP, PDSS2, MT-ND5, G6PC, HADHB, LIPT2, MT-TC, MT-TL1, CAMKMT, DNM1L, TPK1, MTO1, NDUFAF2, LIPT1, MLYCD, MT-TT, MT-ND6, NDUFAF6, PMPCA, MT-TN, GATC, PHKG2, PET100, NDUFAF3, MT-TV, NDUFA1, MT-ND3, SLC25A42, ACAD9, COQ8A, NDUFAF5, BOLA3, MICOS13, UQCRFS1, NDUFS3, MT-CO2, MT-TH, POLG, NDUFB11, CYC1, SUCLG1, ALDOB, LARS2, PCCA, MT-TE, NDUFA6, MT-CYB, COX10, COX6B1, SLC25A4, OGDH, NDUFAF1, PYGL, SURF1, PUS1, EARS2, SLC25A3, MRPS14, ATPAF2, AGK, HADHA, HADH, NADK2, TMEM126B, MIPEP, DGUOK, MRPS16, MT-TQ, NDUFS1, SERAC1, NDUFS8, ATP5F1D, GFER, DLAT, PDHA1, SUCLA2, MT-TW, BCKDHA, FBXL4, PDP1, MPC1, ETHE1, YARS2, GATB, NUBPL, MT-TK, TAZ, UQCC3, MPV17, NDUFB9, NDUFAF8, FBP1, FH, NDUFAF4, ISCU, NDUFS7, ELAC2, BCKDHB, SLC25A26, IBA57, COX8A, RRM2B, QRSL1, FARS2, DLD, TRNT1, DBT, COQ9, RMND1, MT-ND4, TRMU, LRPPRC, CA5A, TK2, PCCB, TSFM, COA8, NDUFA4 |
| HP LACTICACIDURIA | MT-ND4, MT-ND6, SUCLG1, GATM, MT-TW, MT-TL1, MT-ND3, LDHD, MT-ATP6, MT-ND1, PET117, MT-TV, MT-TK, MT-ND2, NDUFB10, MT-ND5, SLC25A21, PDP1 |
| HP SEVERE LACTIC ACIDOSIS | PDHA1, SLC25A26, PDSS2, TRMU, COQ2, MT-TT, MT-TE |

Table S3. The result of univariate Cox proportional hazard model for lactate-related genes in OS and PFS

| Genes | HR 95CI% (OS). | P value (OS) | HR 95CI% (PFS) | P value (PFS) |
| --- | --- | --- | --- | --- |
| GTPBP3 | 1.21(0.84-1.75) | 0.3138 | 1.14(0.82-1.58) | 0.4361 |
| PC | 0.9 (0.74-1.11) | 0.3453 | 0.99 (0.82-1.2) | 0.9477 |
| SLC25A42 | 1.32 (0.99-1.76) | 0.0586 | 1.3(1.01-1.68) | 0.0451 |
| ATAD3A | 0.91 (0.69-1.19) | 0.4848 | 1.05 (0.81-1.35) | 0.7301 |
| HPDL | 1.01 (0.89-1.14) | 0.9094 | 0.91 (0.82-1.02) | 0.0955 |
| ACAT1 | 0.83 (0.63-1.08) | 0.1567 | 0.92 (0.72-1.17) | 0.4947 |
| PNPT1 | 0.94 (0.67-1.33) | 0.7343 | 1.0 (0.76-1.4) | 0.8404 |
| PUS1 | 1.04 (0.75-1.44) | 0.8174 | 1.2 (0.9-1.61) | 0.213 |
| SLC25A4 | 1 (0.73-1.38) | 0.9979 | 0.92 (0.69- 1.23) | 0.5929 |
| CFH | 1.04 (0.91-1.2) | 0.5538 | 1.06 (0.93-1.2) | 0.3725 |
| CFI | 0.97 (0.85-1.12) | 0.7194 | 0.98 (0.87-1.12) | 0.8139 |
| CD46 | 0.98 (0.76-1.26) | 0.8594 | 1.06 (0.85-1.33 | 0.5875 |
| HBB | 1.01 (0.91-1.12) | 0.8459 | 1.06 (0.97-1.16) | 0.2047 |
| MYC | 0.81 (0.65-1.01) | 0.0646 | 0.82 (0.67-1.01) | 0.0575 |
| SPP1 | 1.09 (1.01-1.17) | 0.0333 | 1.08 (1.01-1.15) | 0.0276 |
| FLI1 | 1.05 (0.88-1.25) | 0.5792 | 1.06 (0.9-1.25) | 0.4784 |
| CHEK2 | 0.85 (0.62-1.16) | 0.3021 | 0.86 (0.65-1.14) | 0.2947 |
| IRAK1 | 1.19 (0.89-1.59) | 0.2353 | 1.22 (0.95-1.58) | 0.1177 |
| KCNN4 | 0.98 (0.86-1.12) | 0.7725 | 1.06 (0.93-1.21) | 0.416 |
| ACADM | 0.82 (0.67-0.99) | 0.0444 | 0.86 (0.72-1.03) | 0.1037 |
| COL4A1 | 1.0 (0.9-1.3) | 0.4058 | 1.21(1.02-1.43) | 0.0318 |
| L DHD | 0.99 (0.88-1.12) | 0.8958 | 0.95 (0.86-1.06) | 0.341 |
| PCK1 | 0.92 (0.84-1) | 0.0644 | 0.88 (0.82-0.95) | 0.0014 |
| SLC3A1 | 0.98 (0.89-1.07) | 0.6621 | 0.95 (0.87-1.02) | 0.1709 |
| ALDOB | 0.91 (0.85-0.98) | 0.0142 | 0.94 (0.88-1.01) | 0.0941 |
| ETHE1 | 0.82 (0.65-1.05) | 0.1111 | 0.91 (0.73-1.13) | 0.3864 |
| TAZ | 1.36 (0.99-1.87) | 0.0557 | 1.19 (0.89-1.57) | 0.2369 |
